# Disease phenotypic screening in neuron-glia cocultures identifies blockers of inflammatory neurodegeneration

**DOI:** 10.1016/j.isci.2024.109454

**Published:** 2024-03-08

**Authors:** Timothy J.Y. Birkle, Henriette M.G. Willems, John Skidmore, Guy C. Brown

**Affiliations:** 1 Department of Biochemistry, University of Cambridge, Cambridge CB2 1QW, UK; 2 ALBORADA Drug Discovery Institute, Cambridge CB2 0AH, UK

**Keywords:** Bioinformatics, Neuroscience

## Abstract

Neuropathology is often mediated by interactions between neurons and glia that cannot be modeled by monocultures. However, cocultures are difficult to use and analyze for high-content screening. Here, we perform compound screening using primary neuron-glia cultures to model inflammatory neurodegeneration, live-cell stains, and automated classification of neurons, astrocytes or microglia using open-source software. Out of 227 compounds with known bioactivities, 29 protected against lipopolysaccharide-induced neuronal loss, including drugs affecting adrenergic, steroid, inflammatory and MAP kinase signaling. The screen also identified physiological compounds, such as noradrenaline and progesterone, that protected and identified neurotoxic compounds, such as a TLR7 agonist, that induced microglial proliferation. Most compounds used here have not been tested in a neuron-glia coculture neurodegeneration assay previously. Thus, combining a complex cellular disease model with high-content screening of known compounds and automated image analysis allows identification of important biology, as well as potential targets and drugs for treatment.

## Highlights

• Live-cell stains allow automated labeling of neurons, microglia, and astrocytes
• A high-content compound screen for neuroprotection against LPS finds 29 hits
• Many neuroprotective compounds target steroid, adrenergic, and MAPK signaling
• Multi-dimensional data enables clustering of compounds by overall induced phenotype

## Graphical abstract

**Figure:**
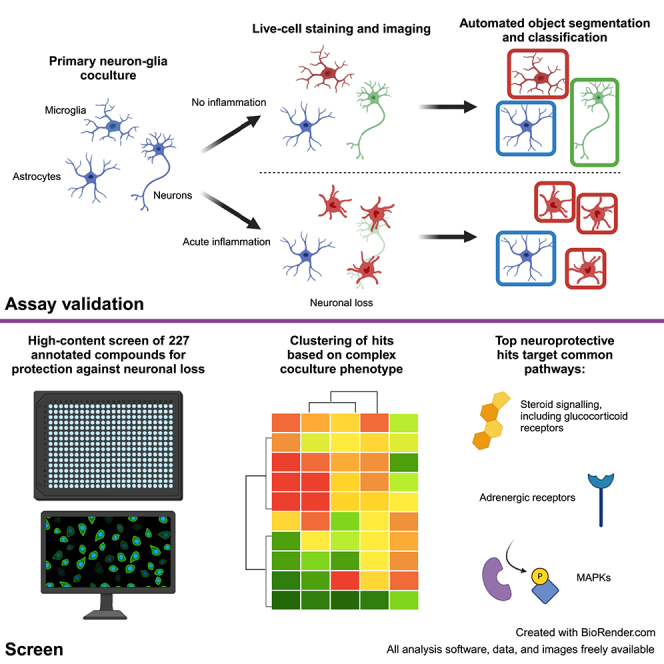

## Introduction

Preclinical translational neuroscience often relies on relatively simple *in vitro* culture systems such as monocultures, and this is particularly true for mid-to high-throughput biology where the simplicity and robustness of experiments and analysis is paramount. However, monocultures of neurons, for example, are of limited utility for modeling neuropathology, as glial cells and their interactions with neurons are important in many neuropathologies, including neurodegeneration.^1^ This lack of realistic disease models at early preclinical stages may contribute to failures when translating results to later stages, and therefore may also contribute to pharmaceutical companies considering drug development for neurodegenerative diseases to be high risk. 2D cultures of primary cells or stem cell-derived cells are at the forefront of what is currently possible to use for screens.^2^^,^^3^ More physiological disease models are available, such as *in vivo* mouse models and 3D cultures/organoids, but these are generally not tractable for screening currently, though there has been some recent progress.^4^^,^^5^^,^^6^

Inflammation from glial cells, and microglia in particular, is now a central topic in neurodegenerative disease research and has been extensively studied and reviewed.^1^^,^^7^ In Alzheimer’s disease (AD), for example, a large proportion of genetic risk for sporadic disease can be attributed to genes that are mostly or exclusively expressed by microglia in the brain.^8^ Microglia are now considered to contribute to diverse brain pathologies including aging, stroke, trauma, autism, schizophrenia, and Parkinson’s disease,^9^^,^^10^^,^^11^^,^^12^ and in culture these cells mediate neuronal loss in response to lipopolysaccharide (LPS), amyloid-β, and tau.^13^^,^^14^^,^^15^^,^^16^ Astrocytes (the other major glial cell type) also regulate neuronal health and disease, and furthermore are necessary for microglial survival *ex vivo* in the absence of specially modified culture media.^17^^,^^18^^,^^19^^,^^20^^,^^21^^,^^22^ As such, mixed cultures of neurons, microglia and astrocytes together are essential for modeling neurodegenerative disease.

However, appropriate microscopy and image analysis techniques are needed for the use of these models. Longstanding methods can extract limited quantitative information out of complex images (for example, levels of a particular protein), yet these images hold a wealth of potentially more functionally relevant information including the distribution, shape, and physical relationships between cell types. Extracting these data would ensure that the increased investment in more sophisticated *in vitro* mixed culture disease models is compensated by increased data and insight from any one experiment. Indeed, complex model systems in neurobiology have generally proven difficult to adapt for screening and a major outstanding challenge is to establish robust high-content image analysis techniques to suit these assays.^3^ There has been progress in recent years, and there are now multiple studies reporting high-content mixed culture assays where individual cell types are distinguished, often using commercially available platforms. In one study, neuron-astrocyte cultures were used to screen 27 compounds for modifiers of Huntington’s disease pathology via the ThermoFisher Cellomics ArrayScan VTI platform, with an approximate throughput of 480 assay wells per batch of primary cells.^23^ Other recent work has used the PerkinElmer Opera Phenix and Harmony software to distinguish neurons, microglia and astrocytes in neuron-glia cocultures, though no screening was carried out.^24^ However, validated open-source pipelines that can achieve similar assays are rare or non-existent in the literature, and high-content neuron-glia coculture assays remain underutilized. Elsewhere, many studies have proposed or used neuron-glia or neuron-astrocyte cultures for high-content screening (HCS) but focused on specific analysis of, for example, synapse density or neurite outgrowth rather than quantifying individual cell types,^25^^,^^26^^,^^27^ and some have lacked exploration of high-content analysis methods in general.^28^^,^^29^ One recent triculture screening study assessed live cell counts and glial area across 28 conditions,^30^ but this does not fully distinguish cell types (least of all neurons) and analysis of glial area confounds changes in glial number with glial morphology.

Image analysis of mixed cultures for quantification of individual cell types consists of discrete steps, the first of which is segmentation of single cell objects for further analysis.^31^ This can be challenging for monocultures but is especially difficult for mixed cultures where cell types with different nuclear morphologies may each require a different segmentation method. Attempting this can result in complicated workflows, which are both difficult to design and unlikely to be particularly robust.^32^ ‘Declumping’ touching cells is another challenge, and such cells are a common feature of neuron-glia cultures. Once identified, cells must be classified to allow information on each cell type to be extracted. Manual thresholding on fluorescent markers is standard but becomes difficult and error-prone when differentiating many cell types, partly because limited availability of channels/markers necessitates the use of low signal-to-noise features such as DNA staining intensity, staining texture, or object shape.

Thankfully, powerful open-source software for both segmentation and classification steps has become widely available in recent years, much of which uses machine learning to deliver impressive accuracy. Such platforms include QuPath,^33^ CellProfiler,^34^ CellProfiler Analyst,^35^ and a host of plugins available to run via these software and the more ubiquitous ImageJ/FIJI,^36^ including Ilastik,^37^ DeepCell,^38^ and Cellpose.^39^ These approaches have already been proven in challenging segmentation and classification tasks elsewhere, including analysis of: protein subcellular localization; dividing nuclear morphology; simple cocultures of two cell lines; and unsupervised, label-free cell classification.^38^^,^^40^^,^^41^^,^^42^^,^^43^^,^^44^^,^^45^

With this in mind, we first aimed to optimize the staining protocol for primary neuron-glia mixed cultures such that they were tractable for mid-throughput screening, and here we show the utility of a recently developed live neuronal stain, NeuroFluor NeuO,^46^ for identifying live neurons. We then demonstrate the accuracy of machine learning-based segmentation and classification approaches for neuron-glia cultures, hosted within the open-source software CellProfiler/CellProfiler Analyst.^34^^,^^35^ Finally, we show that this approach enables HCS for target and drug discovery by screening 227 compounds in primary neuron-glia mixed cultures for neuroprotective effects against inflammatory neuronal loss induced by LPS. LPS induces neuroinflammation via toll-like receptor 4 (TLR4) and is used as a standard neuroinflammatory model,^47^ but LPS is also directly implicated in neurodegenerative disease.^48^^,^^49^^,^^50^ This LPS-induced neurodegeneration assay is well-established, and the loss of neurons has been found to depend on the neurotoxic activity of activated microglia, including reactive oxygen/nitrogen species production and excessive phagocytosis.^14^^,^^51^^,^^52^ Using this model, we find that modulators of steroid, adrenergic and MAP kinase signaling prevent inflammatory neurodegeneration. Furthermore, we generate hypotheses about neuroprotective mechanisms by using the multidimensional data extracted on all cell types in the cultures, which has not previously been possible in such screens. While based on well-accepted methods, our approach is relatively novel in that it combines: (1) a complex, cellular model of disease (LPS-induced neuronal loss in neuronal-glial cultures), (2) automated extraction of information on all cell types, enabling HCS, and (3) an annotated drug library to implicate both drugs and targets in neuroprotection.^53^ We show that this combination enables efficient identification of potential drug therapies, as well as the discovery of novel biology. Moreover, all analysis methods are open-source and the image data from this study is also openly available on BioImage Archive (BIA: S-BIAD890).

## Results

### Staining neuron-glia cultures with NeuO allows optimal visualization

**Figure 1:**
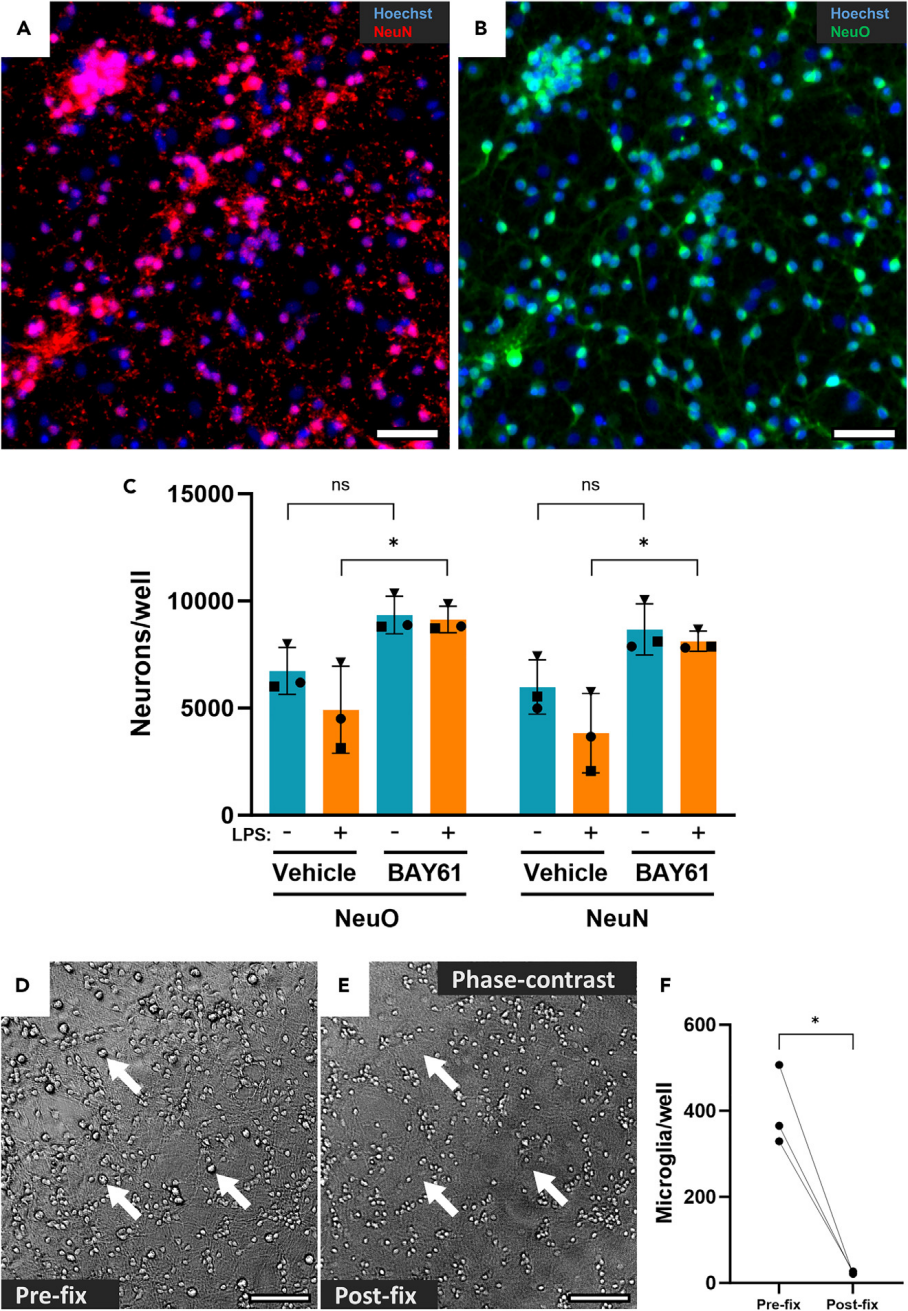
NeuO matches neuron identification by NeuN immunocytochemistry in neuron-glia cultures (A and B) Representative 10x images (cropped) of matching fields-of-view in neuron-glia cultures stained with Hoechst 33342 (nuclei) and α-NeuN ICC (fixed neurons, A) or NeuO (live neurons, B). Scale bars = 50 μm. (C) Average neuronal counts per well (4 images) in cultures treated ± BAY61 (1μM) and ± LPS (100 ng/mL) for 3 days (*DIV10*), quantified from matching fields-of-view with either pre-fix NeuO staining, or post-fix α-NeuN staining. For each of NeuO and NeuN, RM two-way ANOVA with Šídák’s *post-hoc* test. Data are represented as mean ± SD. (D and E) Representative 10x images (cropped) of primary neuron-glia cultures in the transmitted light channel; matching fields-of-view before and after fixation and ICC. Arrows indicate microglia in (D), with equivalent positions marked in (E) to show loss of microglia. Scale bars = 100 μm. (F) Average microglia counts per well before and after ICC of untreated neuron-glia cultures in matched fields-of-view. Paired t-test. **All panels:** single datapoints are each the mean of 3 technical replicates, biological N = 3. ∗p < 0.05. See also Figure S1.

We first aimed to identify the best staining methods for automated image analysis of primary neuron-glia cultures. The gold standard for neuronal identification is immunocytochemistry (ICC) for the neuron-specific antigen NeuN.^54^^,^^55^ However, this requires fixation of the cultures, and does not distinguish between live and dead neurons. We therefore tested staining with NeuroFluor NeuO, as this specifically stains live neurons.^46^ To directly compare NeuO to NeuN, we repeated an experiment from a recent study showing loss of neurons from neuron-glia cultures upon LPS treatment and rescue by the SYK inhibitor BAY61-3606 (hereafter, BAY61).^13^ The same fields-of-view were imaged with both NeuO (before fixing) and NeuN staining (after fixing), allowing direct comparison (Figures 1A and 1B). NeuO staining showed excellent correspondence with α-NeuN labeling, and NeuO-based quantification closely replicated NeuN-based analysis in terms of the relative effects of LPS and BAY61 treatments (Figure 1C). LPS reduced neuron numbers in vehicle-treated wells by 1,810 per image according to NeuO and 2,150 according to NeuN, while BAY61 increased neuron counts in LPS-treated wells by 4,211 according to NeuO and 4,290 according to NeuN. Importantly, either method led to the same experimental conclusion that BAY61 is neuroprotective against LPS (Figure 1C). Lastly, we found no evidence of NeuO strongly staining any cells other than NeuN-positive neurons (Figures 1A, 1B, and S1A).

Despite similar relative neuron counts between conditions, absolute NeuN-based counts were overall slightly but significantly reduced relative to NeuO-based counts of the same cultures (Figure S1B), and further investigation established that the fixation and staining steps resulted in some loss of both total cells and neurons, which may explain this difference (Figures S1C and S1D). Given the risk of cell loss with fixation, we also manually counted microglia in this experiment using their prominence and large size relative to granule neurons in phase-contrast images (Figures 1D and 1E; see Figure S1E for high-magnification comparison and confirmation of this method using IB_4_, which only stains microglia). Strikingly, microglia were nearly completely lost after fixation and ICC of the cultures, preventing analysis of this important cell type (Figure 1F). This microglial loss may be exacerbated by the exposed morphology of microglia in cerebellar cocultures, which may predispose them to being physically washed away with the wash steps of ICC. Going forward, we therefore used NeuO to identify live neurons, together with Hoechst 33342 to stain all nuclei, and IB_4_-AF594 to stain microglia. This combination of fluorescent stains enabled us to distinguish the known cell types in the cultures by eye (Figure S1A) and subsequently proved sufficient for automated classification of the cell types. Astrocytes were identified by their large, oval nuclei (Figure S1A), which is a feature of astrocytes both here and in other coculture models.^29^^,^^56^ Staining with Hoechst and anti-GFAP immunocytochemistry confirmed that these nuclei belong to astrocytes (Figure S1F).

### Machine learning approaches accurately segment and classify nuclei in complex mixed culture images

**Figure 2:**
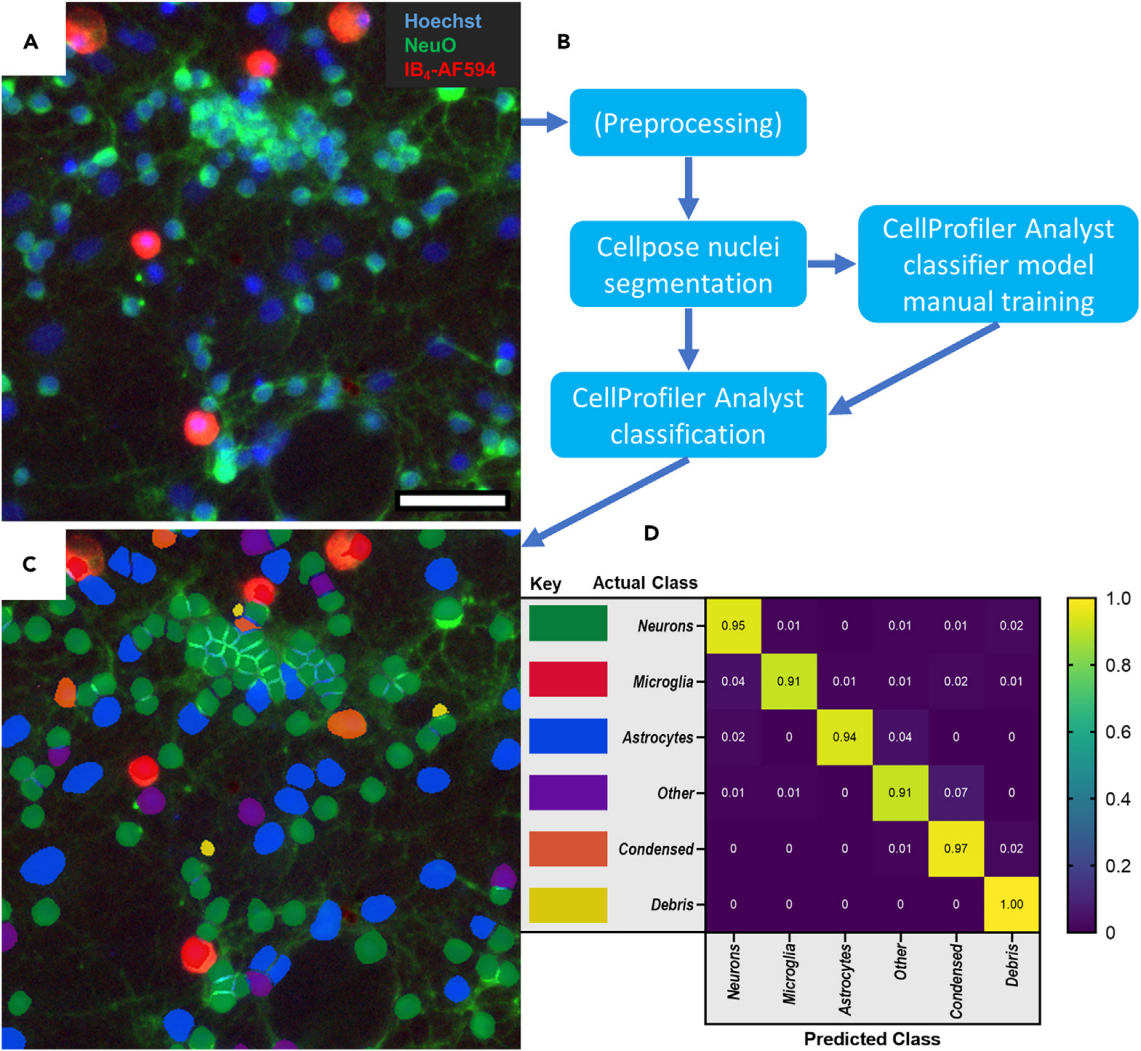
Machine learning-based analysis for accurate, automated cell classification in neuron-glia cultures (A) Representative neuron-glia culture 10x image (cropped) after staining with Hoechst 33342 (nuclei), NeuO (live neurons), and IB_4_-AF594 (microglia). Scale bar = 50 μm. (B) Schematic of the necessary image processing steps required to achieve cell-by-cell classification. (C) Image from (A) with segmentations and classifications overlaid. (D) Heatmap confusion matrix plotting proportion of manually annotated cells of each class (Actual) being predicted to be any given class (Predicted) by a classifier model, during classifier validation on images from experiments separate from the training set. See also Figure S2.

After standard pre-processing of images where necessary, such as the use of background subtraction, automated cell classification requires three broad steps: segmentation, feature measurement for each object, and classification (Figures 2A and 2B). Segmentation defines single-cell objects from an image and is particularly difficult for neuron-glia cultures where diverse cell/nuclear morphologies are present, along with tightly associated groups of neuronal cells. Combined, these features result in segmentation errors, of which over-segmentation and under-segmentation are the most common. (Figures S2A and S2B).

We compared standard segmentation solutions available with freely available software for their accuracy at distinguishing nuclei in challenging neuron-glia culture images: ImageJ,^36^^,^^57^ QuPath,^33^ and CellProfiler.^34^ In addition, we tested a deep-learning-based approach provided by Cellpose,^39^ running within CellProfiler. Each approach was independently optimized for nuclear segmentation, though we limited the ImageJ approach to a basic method for use as a baseline measure. When presented with the same random, background-subtracted images of the Hoechst channel from previous neuron-glia experiments, QuPath and Cellpose outperformed ImageJ and CellProfiler by a large margin (Figure S2C). We selected Cellpose for further use due to the much-reduced over-segmentation errors, which mostly affects the rarer astrocyte population (thus having a disproportionate effect on astrocyte counts), and the ability to implement it as a plugin within CellProfiler as part of a broader image analysis pipeline.

Next, segmented cells are measured for their shape and intensity features across all channels, and finally this data is used for classification. We used a machine learning-based approach via the companion software to CellProfiler, CellProfiler Analyst^35^ (CPA), as machine-learning methods take all object features into consideration (here, 126 in total after exclusion of irrelevant features; Table S1) and can more accurately classify cells than a manual thresholding approach.^38^ The most effective type of models for our data were Random Trees and Support Vector Machines (as compared to other CPA-hosted machine learning-based classification approaches, detailed in the STAR Methods), which were trained to distinguish neuron, microglia, astrocyte, and condensed nuclei, as well as ‘other’ nuclei and debris. ‘Other’ represents a class of rare cells which appear to be dying neurons based on neuronal brightfield morphology, very weak NeuO staining, and Hoechst staining intensity between that of neurons and condensed cells. Manual annotation of a few hundred randomly selected cells of each class was sufficient to achieve highly accurate classification of cells in cerebellar neuron-glia cultures, as validated on images from experiments separate from those used for model training (Figures 2C and 2D). For Random Trees classification, the most useful features included measurements of Hoechst channel intensity, nuclear size, and IB_4_/NeuO intensity (Table S2), confirming that the classification is using the expected features to identify cells. This model was used to identify the neuroprotective effects of Syk inhibition on cerebellar neuron-glia cultures in a recent study,^13^ and was also validated by k-fold cross-validation (k = 5; Figure S2D). We also achieved high performance using the same method for hippocampal neuron-glia cultures, showing that this approach generalizes to neuron-glia cultures from different brain regions (Figure S2E). Overall, machine learning-based segmentation and classification steps produce highly accurate cell identification in complex neuron-glia cultures.

### Design and validation of a high-content screen for neuroprotective compounds

We next aimed to prove the utility of this approach for target discovery using HCS. The screen was designed to assay for neuroprotection against LPS-induced neuronal loss in primary neuron-glia cultures from cerebellum, which we have previously extensively characterised.^13^^,^^14^^,^^51^^,^^52^^,^^58^^,^^59^ LPS induces and models neuroinflammation, and may be directly involved in neurodegenerative diseases.^47^^,^^48^^,^^49^^,^^50^ This inflammatory neuronal loss is dependent on microglia and is therefore a good example of a disease-relevant assay that requires the use of complex cocultures. Moreover, it has not previously been possible to use this or similar model systems in high-content target discovery. To screen, we used an annotated library of compounds with known targets, aiming to identify both neuroprotective drugs and targets based on those drugs’ annotated activities.

**Figure 3:**
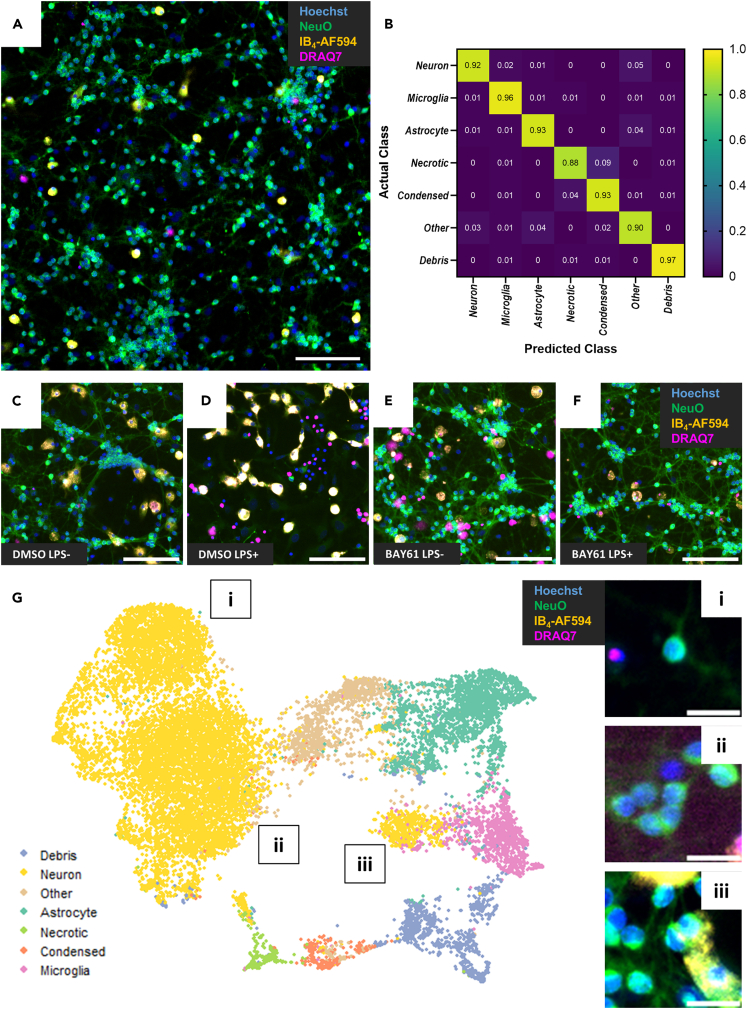
High-content screen imaging and analysis validation (A) Representative 10x image (cropped) of neuron-glia cultures stained with Hoechst 33342 (nuclei), NeuO (live neurons), IB_4_-AF594 (microglia), and DRAQ7 (necrotic cells). Scale bar = 100 μm. (B) Heatmap confusion matrix plotting proportion of manually annotated cells of each class (Actual) being predicted to be any given class (Predicted) by the final trained classifier model from the screen, from 5-fold cross-validation. (C–F) Representative images (cropped) of cultures treated ± LPS (100 ng/mL) and ± BAY61 (1μM) for 3 days (*DIV10*) and stained with Hoechst 33342 (nuclei), NeuO (live neurons), IB_4_-AF594 (microglia), and DRAQ7 (necrotic cells). Scale bars = 100 μm. (G) UMAP plot of the object feature data for 15,710 cells from random negative control wells across all four screen repeats, with retrospective labeling according to the assigned cell type from the screen’s classifier. i, ii, and iii indicate distinct clusters of neurons on the UMAP, which correspond to the inset image tiles (representative neurons from each cluster; scale bars = 10 μm). Key UMAP hyperparameters: n_neighbors = 15, min_dist = 0.1. See also Figures S3–S5.

Compounds were selected from a library with known targets and potencies, as described in detail in the STAR Methods, and were screened at 1μM. Details for all selected compounds can be found in Table S4. Three technical replicates for each compound were included per repeat, with and without LPS, and these were randomly distributed within each plate while ensuring one replicate per compound per plate, again with and without LPS (Figure S3A). Controls were also randomly distributed within each plate and consisted of DMSO (negative control) and BAY61 (1μM, positive control for neuroprotection against LPS) represented six times per plate with and without LPS. Treatments were added topically to cultures in 384-well plates at 7 days *in vitro*, then NeuO, Hoechst 33342, IB_4_-AF594 and DRAQ7 were topically added at 10 days *in vitro* for staining (Figure 3A). DRAQ7 is a cell-impermeant DNA stain that therefore only stains nuclei of necrotic cells, and was included in the screen (unlike in the earlier methods development) due to the availability of a far-red filter channel on the high-content microscope. 4 images were captured per well at 10× objective magnification on an IN Cell Analyzer 6000 instrument, with fields-of-view collectively covering ∼80% of each well and capturing roughly 10,000 neurons. Though the cells were live, this was an endpoint assay and each field-of-view was only imaged once, avoiding complications from phototoxicity with repeated imaging. Four biological replicates of the screen were conducted using four distinct culture preparations. All images are available on BioImage Archive (BIA: S-BIAD890) for replication of the following analysis or for use in any other analysis.

Image analysis was performed with the approach optimized above, using Cellpose for accurate segmentation of nuclei within a CellProfiler pipeline that produced measurements of over 100 different features for each object (Table S1). Cells used for training were picked equally from all four repeats and thereafter sampled randomly from all treatment conditions. The final training data consisted of 11,274 cells, balanced between LPS-untreated and LPS-treated wells (5,692 and 5,582 cells, respectively) and across the different cell types (approximately 1,600 cells per class). The resulting classifier achieved high accuracy for all cell types including >92% recall (the proportion of actual cells of each type being correctly classified) for the three major cell types (neurons, microglia, and astrocytes; Figure 3B), and prioritized the expected cell features for cell type discrimination including percentile/mean intensity measurements of each stain and the size of the nuclei (Table S3). As this new classifier was trained on 4-colour images which included DRAQ7, necrotic cells could be differentiated from condensed (likely apoptotic) cells unlike in the previous methods development. Images of control culture wells were as expected, with strong neuronal loss induced by LPS and strong neuroprotection provided by BAY61 (Figures 3C–3F). The classifier maintained high accuracy for each of the four control conditions separately (DMSO ± LPS, BAY61 ± LPS), validating the analysis for cultures with phenotypes ranging from extreme neurodegeneration to complete protection (Figure S3B).

We next validated that the chosen cell type classifications reflect the true variety of cells visible in our cultures when using the chosen staining method. Uniform manifold approximation and projection (UMAP) analysis of the single-cell level object feature data (127 dimensions; Table S1) for 15,710 cells from random negative control wells across all four screen repeats successfully segregated in two dimensions the cell types that had been identified by eye in the images (Figure 3G). The boundaries of these clusters corresponded well with the classifications that had been assigned by the analysis pipeline, these in turn being an accurate reflection of the original manual training, and furthermore did not vary between biological repeats (Figure S4). The most important cell features identified by the trained classifier also clearly distinguished cell types in the dimensionality reduction (Figure S4). Notably, neurons appeared in three clusters (Figure 3G); on analysis of the corresponding cells, it became clear that these were neurons which were isolated from other cells (Figure 3G, cluster i), adjacent to other cells/neurons (thus having additional Hoechst staining at their border; Figure 3G, cluster ii), or adjacent to a microglia (thus having some IB_4_ staining; Figure 3G, cluster iii). Accordingly, these neuronal groups were clearly distinguished by both Hoechst intensity measurements taken specifically at the edge of cell objects and IB_4_ intensity measures (Figure S4). This ability to distinguish neurons that differ only in their immediate proximity to other cells reflects the richness of the object data generated by CellProfiler, but these groupings are unlikely to indicate neuronal subtypes. Overall, the labels applied during classification were found to correspond with genuinely distinct cell types in the original imaging data.

### Quality control and data normalization

For quality control, we first visualized plate-to-plate variation in LPS-untreated DMSO wells by using principal component analysis on the full data for all cell types (PCA; Figure S5A). Outlier analysis of the cell type counts (ROUT, Q = 1%) highlighted one plate in the second repeat that had higher necrosis and 25% lower neuron counts than other plates in that repeat, so this plate was subsequently excluded. One plate of the fourth repeat was flagged due to a small increase of condensed cells (∼30 cells per frame), but with no change in any other cell counts the data from this plate was retained. All plates’ responses to LPS were similar overall (Figure S5B).

Cell counts did vary between repeats, likely due to variable plating densities (Figures S5A and S5C). To minimize the effect of this variation on the analysis, count data for the foremost cell types analyzed in this study (neurons and microglia) were Min-Max normalized using the LPS-untreated and LPS-treated repeat-wise median counts. This was possible because of the large increase in microglial numbers and large decrease in neuronal numbers after LPS treatment (Figures S6A–S6D), and for neuron counts the Min-Max normalization successfully reduced the average per-condition coefficients of variation by 32% (Figures S6A and S6B). Though there was some variation in cell type proportions between repeats, the relative numbers of neurons and microglia (the two most important cell types for this assay) were similar (within ∼10%; Figure S5D).

Data from all conditions was used to assess per-row and per-column variation, and small trends were observed in both cases (Figures S5E–S5L). However, any effects were minor and treatment well locations had been randomized. We confirmed that this randomization was effective by analyzing average row and column locations for each treatment and observed no difference between compounds subsequently found to be neuroprotective and non-hits (Figures S5M and S5N). In a per-well analysis, no individual wells were identified as strong outliers across the screen (Figures S5O and S5P), and any small effects of well location would have been similarly mitigated by randomized treatment locations. Overall, given the small size of any plate location effects and the mitigation by randomization of treatment layouts, no further corrections were made for row, column, or well effects.

Non-normalized data for all cell types are presented in Figure S6. LPS treatment caused loss of 90–95% of the live neurons (Figure S6A), increased the number of necrotic cells about 10-fold (Figure S6F), and increased the number of microglia 2- to 3-fold (Figure S6C), but did not change the number of astrocytes (Figure S6E). After normalization, the neuroprotection assay achieved an overall Z′-factor of 0.927 between DMSO-treated wells in the presence and absence of LPS, and 0.724 between DMSO- and BAY61-treated wells in the presence of LPS. The strong neurodegeneration induced by LPS in the screen, as compared to some of our previous data (for example, Figure 1) may be explained by the optimized LPS preparation protocol developed for the screen, where the LPS was sonicated prior to use, potentially dispersing the LPS-containing micelles and increasing the effective concentration of LPS available to activate microglia. Different gas exchange or the different plate bottom material in the screening plates may also have contributed to the increased neuronal loss induced by LPS in the screen.

### Compounds protecting against LPS particularly affect steroid signaling, adrenoreceptors, and MAP kinases

**Figure 4:**
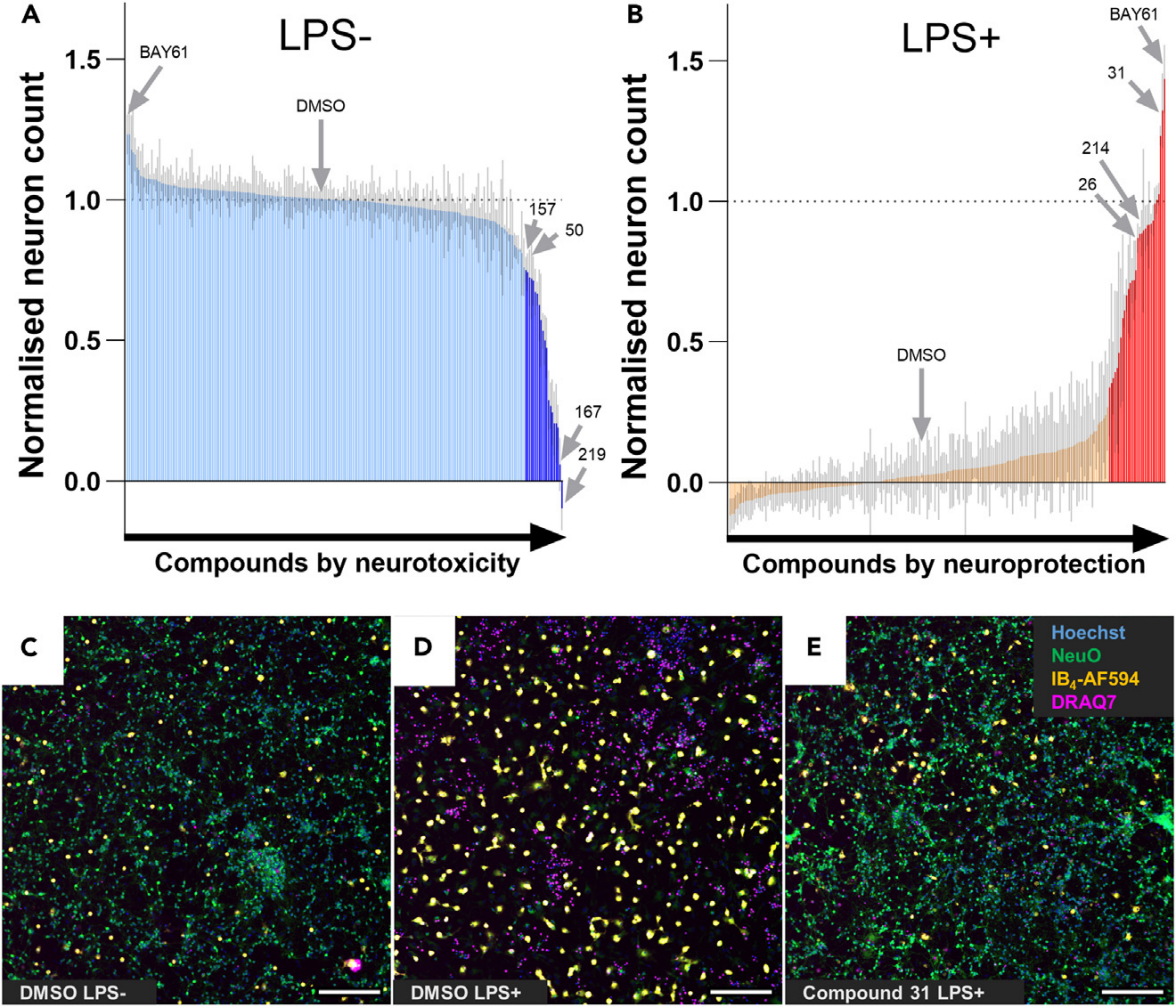
The screen identifies neurotoxic compound in the absence of LPS, and neuroprotective compounds in the presence of LPS (A and B) Average normalized neuron counts from neuron-glia cultures treated without (A) and with (B) LPS and each compound in the screen, and ordered by increasing neurotoxicity or neuroprotection respectively. RM two-way ANOVA with Dunnett’s *post-hoc* test comparing all treatments to DMSO in the presence and absence of LPS. Darker regions of each graph indicate p < 0.05 significance. Data are represented as mean ± SD, N = 4. (C–E) random 10x images from DMSO-treated LPS-untreated, DMSO-treated LPS-treated, and hit compound-treated (compound 31) cultures at *DIV10* after 3 days treatment with 100 ng/mL LPS and staining with Hoechst 33342 (DNA), NeuO (live neurons), IB_4_-AF594 (microglia), and DRAQ7 (necrotic cells). Scale bars = 100 μm. See also Figures S6 and S7.

Average normalized neuronal count data from all repeats (N = 4) was analyzed to identify compounds causing a significant decrease or increase of live neurons versus DMSO within LPS- untreated and LPS-treated wells, respectively (Figures 4A and 4B). Significant compounds from this analysis are therefore neurotoxic or neuroprotective, respectively, and the latter were deemed ‘hits’. In total, 29 compounds were identified as hits, in addition to the positive control (BAY61, an SYK inhibitor), which strongly protected cultures as expected (Table 1). One hit was another SYK inhibitor (compound 26), corroborating our previous finding that SYK inhibitors are neuroprotective in this model of inflammatory neuronal loss.^13^ The TLR4 inhibitor TAK-242/CLI-095/resatorvid (compound 214) also significantly protected, which is to be expected based on LPS activating microglia via this receptor. Images of cultures treated with all hit compounds were manually checked, which confirmed the neuroprotection reported by the analysis (Figures 4C–4E; see Figure S7 for randomly selected images from all hit compound cultures).

**Table 1:** Compound and targets protecting against LPS-induced neuronal loss

ID	ChEMBL ID	Predicted (LS) mean diff.	95% CI of diff.	Summary	Adj. p value	Name	Targets														Label	Cluster
BAY61	CHEMBL1242100	−1.411	−1.659 to −1.164	∗∗∗∗	<0.0001	BAY61-3606	*SYK*														Other	3
31	CHEMBL1451	−1.298	−1.546 to −1.050	∗∗∗∗	<0.0001	Triamcinolone	*NR3C1*^*a*^	NFKB1	NFE2L2												Steroid	3
105	CHEMBL603	−1.208	−1.456 to −0.9603	∗∗∗∗	<0.0001	Zafirlukast	CYSLTR1	CYSLTR2	*MAPK14*												MAPK	3
29	CHEMBL1399	−1.001	−1.249 to −0.7530	∗∗∗∗	<0.0001	Anastrozole	CYP19A1														Steroid	2
67	CHEMBL277535	−0.9812	−1.229 to −0.7335	∗∗∗∗	<0.0001	Bifonazole	CYP17A1	CYP51A1	CYP3A4												Steroid	2
28	CHEMBL1358	−0.966	−1.214 to −0.7183	∗∗∗∗	<0.0001	Fulvestrant	PGR	ESR1	ESR2	ESRRA	EPHX2	NR1H4									Steroid	2
139	CHEMBL373250	−0.9072	−1.155 to −0.6594	∗∗∗∗	<0.0001	L-838417	GABRA3 ^*a*^	GABRB3 ^*a*^	GABRG2 ^*a*^	GABRA2 ^*a*^	GABRA1 ^*a*^	GABRA5 ^*a*^									Other	2
77	CHEMBL36	−0.8951	−1.143 to −0.6473	∗∗∗∗	<0.0001	Pyrimethamine	DHFR	SLC47A1													Other	2
114	CHEMBL717	−0.8911	−1.139 to −0.6434	∗∗∗∗	<0.0001	Medroxyprogesterone Acetate	PGR ^*a*^	AR ^*a*^	*NR3C1*^*a*^	AKR1C3	ESR1 ^*a*^	ESR2 ^*a*^									Steroid	2
30	CHEMBL1437	−0.8901	−1.138 to −0.6423	∗∗∗∗	<0.0001	Norepinephrine	*ADRA1A*^*a*^	*ADRA1D*^*a*^	*ADRA2A*^*a*^	*ADRA2B*^*a*^	*ADRA2C*^*a*^	*ADRB3*^*a*^	ADRB1 ^*a*^								ADR	1
109	CHEMBL679	−0.8781	−1.126 to −0.6304	∗∗∗∗	<0.0001	Epinephrine	*ADRA2A*^*a*^	*ADRA2B*^*a*^	*ADRA2C*^*a*^	*ADRA1A*^*a*^	ADRA1B ^*a*^	*ADRA1D*^*a*^	*ADRB3*^*a*^	ADRB2 ^*a*^	CA1 ^*a*^						ADR	1
161	CHEMBL2204502	−0.871	−1.119 to −0.6232	∗∗∗∗	<0.0001	XL888	HSP90AA1	HSP90AB1													Other	3
111	CHEMBL707	−0.8618	−1.110 to −0.6140	∗∗∗∗	<0.0001	Doxazosin	*ADRA1B*	*ADRA1D*	*ADRA1A*	SLC6A3	HTR2B	*ADRA2C*	HTR2C	*ADRA2A*	HTR4						ADR	2
214	CHEMBL426184	−0.8595	−1.107 to −0.6117	∗∗∗∗	<0.0001	TAK-242/CLI-095/resatorvid	TLR4														Other	2
152	CHEMBL3672369	−0.8444	−1.092 to −0.5966	∗∗∗∗	<0.0001	OTS964	PBK	NEK1													Other	3
26	CHEMBL1235110	−0.7291	−0.9769 to −0.4814	∗∗∗∗	<0.0001	NA	*SYK*	*MAPK1*	MAPK3	GP6											MAPK	2
24	CHEMBL41	−0.6942	−0.9419 to −0.4464	∗∗∗∗	<0.0001	Fluoxetine	SLC6A4	*ADRA2A*	HTR1A ^*a*^	HTR1B ^*a*^	HTR1D ^*a*^	HTR1F ^*a*^	CYP2C19	HTR2A ^*a*^	HTR2C ^*a*^	SLC6A2	*ACHE*	NET	SIGMAR1	CYP2D6	Other	2
130	CHEMBL379225	−0.6927	−0.9404 to −0.4449	∗∗∗∗	<0.0001	N-(4-(1,1,1,3,3,3-hexafluoro-2-hydroxypropan-2-yl)phenyl)-N-methylbenzamide	NR1H3 ^*a*^	NR1H2 ^*a*^													Steroid	1
22	CHEMBL1093059	−0.6838	−0.9315 to −0.4360	∗∗∗∗	<0.0001	NA	NOS2	KEAP1	NQO1 ^*a*^												Other	1
165	CHEMBL1088752	−0.662	−0.9097 to −0.4143	∗∗∗∗	<0.0001	Losmapimod	*MAPK14*	STK24	*MAPK11*	MAPKAPK2											MAPK	1
14	CHEMBL1232461	−0.6431	−0.9466 to −0.3395	∗∗∗∗	<0.0001	NA	BRD4	BRD3	BRD2	BRDT											Other	3
191	CHEMBL577784	−0.5855	−0.8332 to −0.3377	∗∗∗∗	<0.0001	BX-795	TBK1	IKBKE	CGAS	PDK1	ULK1	ULK2	PDPK1								Other	1
42	CHEMBL1914489	−0.559	−0.8068 to −0.3113	∗∗∗∗	<0.0001	AZD1981	PTGDR2														Other	1
119	CHEMBL119385	−0.4899	−0.7376 to −0.2421	∗∗∗∗	<0.0001	Neflamapimod	*MAPK11*	MAPK12	MAPK13	*MAPK14*	ABL2										MAPK	1
112	CHEMBL709	−0.4362	−0.6840 to −0.1885	∗∗∗∗	<0.0001	Alfuzosin	*ADRA1B*	*ADRA1D*	*ADRA1A*	*ACHE*											ADR	1
96	CHEMBL507361	−0.3815	−0.6292 to −0.1338	∗∗∗∗	<0.0001	PD-0325901	*MAP2K1*	BRAF	*MAP2K2*	*MAPK1*	MAP2K5										MAPK	3
6	CHEMBL1200323	−0.3681	−0.6158 to −0.1203	∗∗∗∗	<0.0001	Labetalol Hydrochloride	ADRB1	ADRB2	ADRB3	*ADRA1A*	*ADRA1B*	*ADRA1D*	*ADRA2A*	ADRA2B	*ADRA2C*						ADR	1
210	CHEMBL2103875	−0.3457	−0.5934 to −0.09795	∗∗∗	0.0003	Trametinib	*MAP2K1*	*MAP2K2*	ABCB1												MAPK	1
20	CHEMBL3798846	−0.3284	−0.5761 to −0.08065	∗∗∗	0.0008	OICR-9429	WDR5														Other	1
79	CHEMBL385517	−0.312	−0.5597 to −0.06426	∗∗	0.002	Saxagliptin	DPP4	DPP9	DPP8												Other	1

Related to Figure 4. Ordered by decreasing effect size. Predicted (LS) mean diff. = mean normalized neuron count after DMSO LPS+ treatment minus after compound LPS+ treatment. −1 represents full protection back to LPS- neuron levels. **Targets:** Gene symbols of known targets for each compound extracted from ChEMBL, with addition mechanism of action (MOA) information. Default MOA is inhibition; suffixed ^a^ indicates agonism. *Italicized* MOAs are those which are repeated amongst all neuroprotective compounds (e.g., NR3C1 is agonised by compounds 31 and 114). **Label:** Terms indicate association of targets with common pathways of interest among the hit data. Steroid = steroid synthesis/signaling modulation; ADR = adrenoreceptor modulation; MAPK = mitogen-activated protein kinase inhibition. **Cluster:** Terms indicate the cluster of the overall hit phenotype as analyzed in Figure 6.

Analysis of the hits revealed that multiple hit compounds targeted proteins of the same class/in the same pathways (as detailed in Table 1). For example, 6 hits targeted receptors/enzymes involved in steroid hormone synthesis and signaling, with many of these compounds being among some of the most protective hits seen. Meanwhile, 5 hit compounds targeted members of the adrenoreceptor family (ADRs) and 6 inhibited MAPK family members.

### Analysis of LPS-untreated cultures identifies neurotoxic compounds

While identification of neuroprotective compounds and targets was the primary goal of our approach, toxicology data is also essential for guiding therapeutic development, and may give insight into neuronal biology. Analysis of neuronal counts identified 20 compounds that significantly decreased the number of live neurons in the absence of LPS (Figure 4A; Table S5). The most neurotoxic compound was rotenone (219), widely used to model Parkinson’s disease.^60^ Rotenone is an inhibitor of mitochondrial complex I, and its neurotoxicity confirms that neurons cannot survive without mitochondrial respiration.^61^ Calcium channel inhibitors were also neurotoxic, though previous studies indicate that inhibitors of some calcium channels can be protective.^62^ Neurotoxicity was also induced by inhibitors of the DNA damage response proteins TDP1 and ATM (167 and 50, respectively), indicating that DNA repair is essential for neuronal survival in culture. The TLR7 agonist vesatolimod (157) was also partially neurotoxic, which may be due to a strong microglial response as indicated by the 6-fold increase in microglial numbers in the absence of LPS (Figure S4B; Table S6). TLR7 recognizes single stranded RNA, particularly of viruses, so this finding suggests that viruses known to affect cognitive function (such as COVID-19) could induce neuroinflammation and neuronal loss by these means. Overall, this secondary data from the screen represents valuable toxicology data for translational research but may also suggest important neurodegenerative biology.

Conversely, some compounds appeared to increase baseline neuronal counts, similar to the previously published effect of the positive control, BAY61 (Figure 4A). However, none of these baseline protective effects were significant here.

### Parallel analysis of microglia suggests mechanisms in primary screening

**Figure 5:**
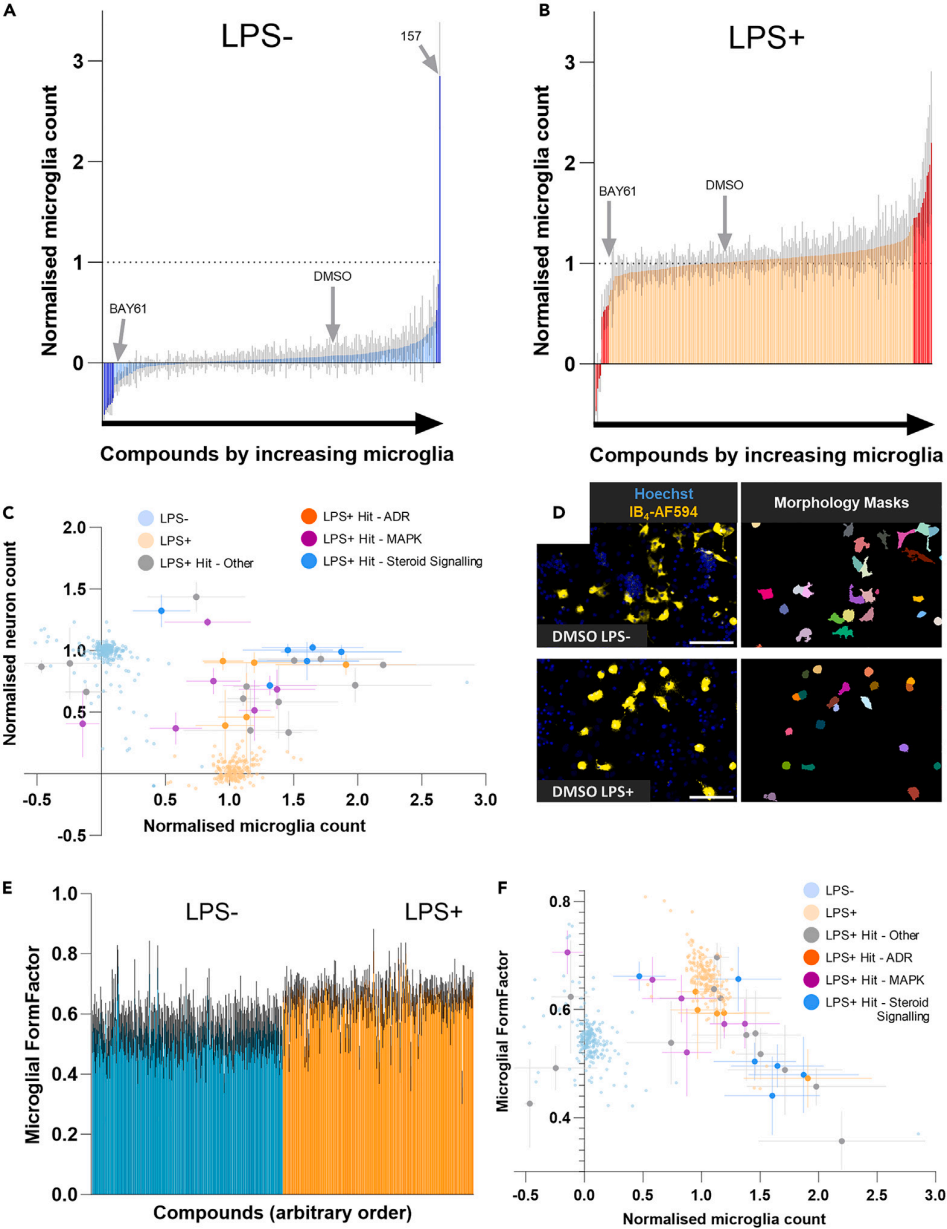
High-content analysis allows interrogation of additional cell types for secondary outcome data (A and B) Average normalized microglia counts from neuron-glia cultures treated without (A) and with (B) LPS and each compound in the screen, and ordered by increasing microglia count. RM two-way ANOVA with Dunnett’s *post-hoc* test comparing all treatments to DMSO in the presence and absence of LPS. Darker regions of each graph indicate p < 0.05 significance. Data are represented as mean ± SD, N = 4. (C) Average normalized neuron counts plotted against average normalized microglia counts for all compounds ± LPS, with LPS-treated data from hit compounds highlighted and colored by target class. Data are represented as mean ± SD, N = 4. (D) Representative Hoechst-IB_4_ labeled images (cropped) and corresponding segmented microglial masks from cultures treated with DMSO for 3 days (*DIV10*) in the presence and absence of LPS, illustrating the microglial morphology analysis. Scale bars = 100μm. Mask colors are arbitrary. (E) Average microglial FormFactor (circularity) from neuron-glia cultures treated ± LPS and ± each compound in the screen. Data are represented as mean ± SD, N = 4. (F) Average microglial FormFactor plotted against average normalized microglia counts for all compounds in the presence of LPS, with data from hit compounds highlighted and colored by target class. Data are represented as mean ± SD, N = 4. See also Figures S6–S8.

A key advantage of our approach is the parallel analysis of all cell types in the cultures. Astrocyte numbers were largely unchanged with LPS (Figure S6E). In contrast, microglia numbers increased with LPS, as expected (Figure S6C), and many treatments altered microglial numbers (Figures 5A and 5B; Tables S6 and S7). Microglia are crucial for LPS-induced neuronal loss, as removal of microglia is sufficient to prevent the loss.^13^^,^^14^ Interestingly, most treatments altering microglial numbers in LPS-treated wells were neuroprotective, regardless of whether microglia increased or decreased (Figure 5C). As LPS induces microglial proliferation, a treatment effect on microglial number may indicate an effect on microglial response to LPS. However, compounds may affect microglial proliferation independent of LPS-induced signaling pathways. Hits modulating ADRs protected neurons without affecting microglia number (Figure 5C), suggesting no effect on microglial proliferative state. Meanwhile, some MAPK inhibitors (and other compounds) dramatically reduced microglial numbers while protecting neurons, clearly blocking LPS-induced proliferation either directly or indirectly. Other MAPK inhibitors had little or no effect on microglial numbers, suggesting dependence on the specific kinases targeted by each compound. Finally, compounds targeting steroid signaling tended to increase microglial numbers in the presence of LPS (but not in its absence, Table S6) despite their strong neuroprotective effects, implying an effect on microglial polarization. Many other hit compounds outside of these categories acted similarly, and these differences are also reflected in representative images (Figure S7).

In order to gain more insight into whether the compounds were affecting microglial state, we analyzed microglial morphology ± LPS in the cultures. It is not possible to define microglial state based on microglial morphology alone, and particularly not in 2-dimensional culture; however, microglial morphology in these cultures does change with LPS treatment and may therefore loosely indicate microglial state. Microglial shape was inferred from microglial cell masks obtained with the IB_4_ staining, and results were improved considerably by use of the accurately classified microglial nuclei for marker-based segmentation of those masks, particularly in microglia-dense images. The FormFactor measurement, equivalent to ‘circularity’ in ImageJ and ranging between 1 (perfectly circular) and 0, gave the best signal-noise ratio for detecting the microglial morphology change induced by LPS. Microglial FormFactor was highly variable in LPS-untreated wells, and microglia converged on a rounder and more ameboid shape with LPS activation (Figures 5D, 5E, and S8). Hit compounds frequently altered microglial morphology in the presence of LPS. Notably, those hit compounds that increased the number of microglia in the presence of LPS (such as compounds affecting steroid metabolism/signaling) tended to decrease the microglial FormFactor back toward (or beyond) that of LPS-untreated microglia (Figure 5F). This suggests that these neuroprotective compounds inhibited the LPS-induced morphological changes of microglia, despite increasing microglial numbers.

### Multidimensional phenotypic data prioritizes hit compounds based on overall disease phenotype

To meaningfully aggregate the multidimensional data output and more closely examine the potential mechanisms of different hit compounds, we hierarchically clustered hits based on their range of output measures taken together (Figure S9A). This analysis aimed to identify groups of compounds that induced similar overall phenotypes in the cultures (with respect to counts for all cell types and microglial morphology) in both the presence and absence of LPS, which was beyond the scope of the previous two-dimensional analyses (Figure 5). Collated cell count and microglial morphology data for all treatment replicates in the screen is available in Table S8.

**Figure 6:**
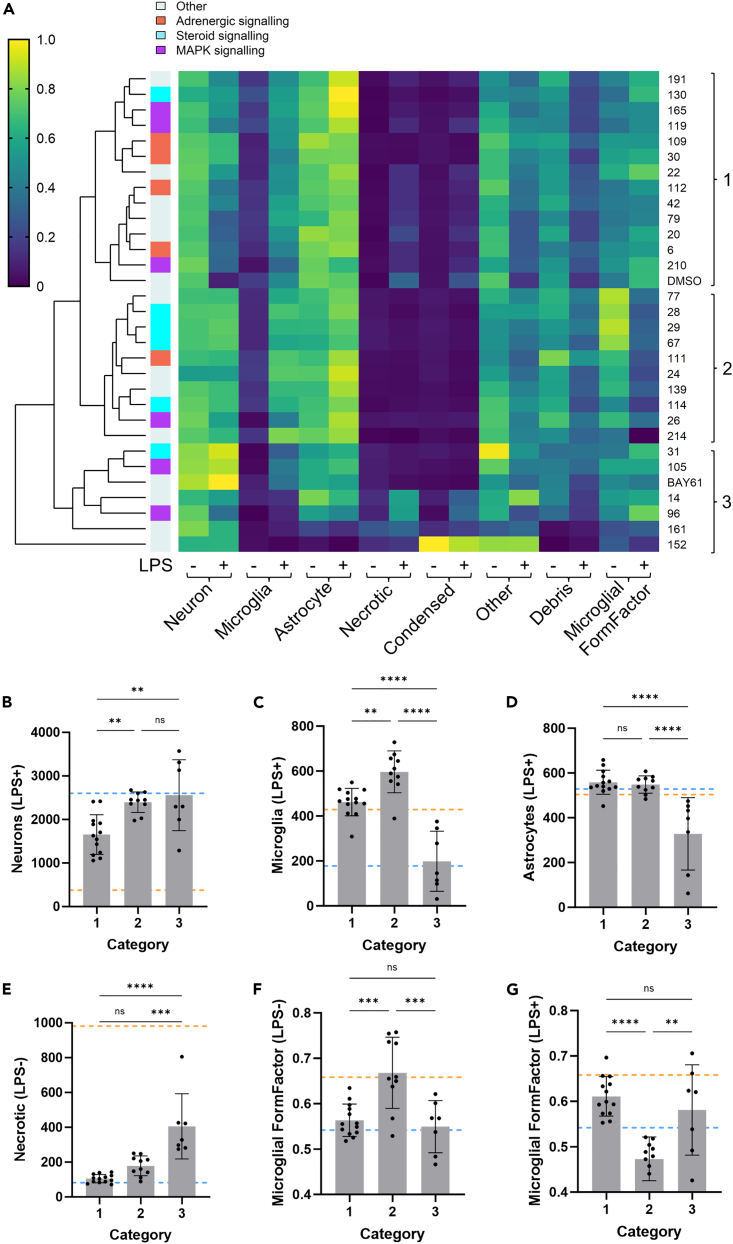
Clustered heatmap analysis of all screen data reveals 3 distinct mechanisms of neuroprotection (A) Clustered heatmap of data from neuroprotective hits and DMSO (counts of each cell type and microglial morphology in the absence and presence of LPS). E.g. “Neuron –“ and “Neuron +” columns display mean neuron counts in the absence and presence of LPS respectively. In the analysis of all compounds (Figure S9A), each data type was min-max normalized between 0 and 1 based on min and max values across both LPS-treated and LPS-untreated conditions to ensure comparability, and this data was then filtered for hits and DMSO. Legend and left-hand side color labels indicate the target pathway for each hit. Compound IDs and category numbers provided on the right-hand side. (B–G) Average data (variable: see Y axes) for hit compounds of each category. Each datapoint is the average value for one hit compound over all screen repeats. Categories 1, 2, and 3 included 13, 10, and 7 hit compounds respectively. One-way ANOVA with Tukey’s *post hoc* test. Blue and orange dashed lines indicate the average data value in LPS-untreated and LPS-treated DMSO wells respectively. Data are represented as mean ± SD. ∗∗p < 0.01, ∗∗∗p < 0.001, ∗∗∗∗p < 0.0001. See also Figure S9.

We focused our analysis on the neuroprotective compounds, within which three broad phenotypes were apparent in the data (Figure 6A). The relevance of these categories is supported by significant differences between them in various dimensions of the data (Figures 6B–6G). However, it should be noted that this analysis will lack the nuance to capture differences in phenotype that are not measured in the original analysis. As displayed at the top of Figure 6A, one group of hit compounds (hereafter, category 1) clustered closer to the negative DMSO control than the rest and gave the weakest neuroprotection of around 57% on average (Figure 6B). These compounds did little to alter glial numbers or microglial morphology (Figures 6C, 6D, 6F, and 6G) and may therefore be inhibiting specific neurotoxic pathways rather than, for example, preventing broadly neurotoxic states of glia. Consistent with this, compound 22 in this group is a NOS2 inhibitor – nitric oxide production is a specific neurotoxic mechanism previously identified in mixed culture.^52^^,^^63^ Other neuroprotective hits in this category include the majority of the adrenergic receptor modulators, and some MAPK inhibitors.

By contrast, in the middle of the Figure 6A there is a more strongly protective cluster of compounds (hereafter category 2; average of around 91% protection; Figure 6B) that increased microglial numbers in the presence of LPS by 39% on average and altered microglial morphology (Figures 6C–6G). These hits may be directly affecting microglial states or polarization and thereby protecting against LPS-induced neuronal loss. Supporting this hypothesis, compound 214 in this group is the TLR4 inhibitor TAK-242/CLI-095/resatorvid, which directly antagonizes LPS-induced signaling and microglial activation via TLR4. We would expect TLR4 inhibition to prevent microglia activation by LPS, supporting the notion that other hits in this cluster may be neuroprotective by altering microglial state. Notably, this cluster also includes most of the steroid signaling-related hits and has little representation of adrenergic signaling-related compounds.

Finally, at the bottom of the Figure 6A there is a cluster of hits (hereafter, category 3) that include the most strongly neuroprotective compounds of the screen and the positive control BAY61 as well as some less effective hits (average of 98% protection; Figure 6B). The most striking feature of this cluster is a decrease in microglial and/or astrocyte numbers in both the presence and absence of LPS (average of 50–60% microglial depletion either ± LPS, average of 35% astrocyte depletion either ± LPS; Figures 6C, 6D, S9B, and S9C), and an increase in necrotic cells (average increase of 400% in the absence of LPS; Figure 6E), which are probably glial cells given that live neurons increased in number. Glial depletion, particularly of microglia, is known to protect neurons against LPS in mixed cultures,^13^^,^^14^ so these compounds probably protect neurons by depleting glia. The most strongly glia-depleting hit compounds 152 and 161 are OTS964 (a PBK/NEK1 inhibitor) and XL888 (a HSP90 inhibitor) respectively.

## Discussion

### Summary of findings

In this study, we demonstrate that complex neuron-glia mixed cultures are suitable for high-content assays using NeuO staining^46^ and machine learning-assisted, automated image analysis.^34^^,^^35^^,^^39^ These methods are most applicable to phenotypic high-content screens (that is, screens identifying modifiers of functional phenotypes, rather than modulators of specific proteins), but can aid low-throughput work as well.

As a proof of concept, we present results from a phenotypic high-content screen aiming to identify compounds (and their cellular targets) that have neuroprotective activity against LPS-induced neuronal loss in primary neuron-glia cultures. We tested 227 compounds over four biological repeats in a screen that included approximately 24,000 image sets and classified 100 million cells. This achieved an overall Z′-factor in the primary neuroprotective assay of 0.927 for DMSO LPS- against DMSO LPS+, 0.724 for DMSO LPS+ against positive control LPS+, and a robust Z-factor of 0.448 for all data taken together. All images are available as a resource at BioImage Archive (BIA: S-BIAD890) and associated analysis pipelines are also available (GitHub: https://github.com/timjyb/Birkle-et-al-2023-HTS). The screen found 29 significantly neuroprotective compounds, of which 14 (and the positive control SYK inhibitor, BAY61) rescued neurons by at least 75%, and a further 8 rescued by at least 50% (Table 1). Some hits reflect the known biology of LPS-induced neuronal loss in these mixed cultures, including: a second SYK inhibitor that protected by 73% (compound 26), the TLR4 inhibitor TAK-242/CLI-095/resatorvid that protected by 86% (compound 214), and an inhibitor of NOS2 that protected by 68% (compound 22). This confirms that the screening method can recapture previously published findings within this model system.^13^^,^^52^ Inflammatory neuronal loss in this model has previously been shown to depend on microglia, hence the particular focus on microglia in the follow-up analysis. Data from LPS-untreated wells was also valuable in identifying neurotoxic compounds, including expected compounds such as rotenone (Table S5). It would be interesting to study these neurotoxic compounds further in neuronal monocultures to determine whether they act directly on neurons or via glia, but this was not investigated here.

The hit compounds were assessed for overlap with respect to targets and pathways, and there were found to be multiple hits affecting steroid signaling, adrenergic signaling, and MAPK signaling. The multidimensional data output from the analysis allowed clustering of neuroprotective hits into 3 general categories: 1) compounds weakly protecting neurons without affecting glia, perhaps by inhibiting specific neurotoxic mechanisms (e.g., the NOS2 inhibitor, compound 22); 2) compounds strongly protecting neurons while modulating microglial number and morphology, suggesting that their protection was by affecting microglial state (e.g., the TLR4 inhibitor, compound 214); and 3) compounds protecting neurons while depleting microglia and/or astrocyte populations (though microglial state may also be affected; e.g., the positive control, BAY61). Category 2 may be particularly relevant for further investigation, as modifying microglial state may be a powerful means by which to influence neuroinflammatory disease progression.^64^ Category 1 compounds/targets may be of lesser interest due to weaker effect sizes, and category 3 compounds/targets may be less promising targets for therapies given the possible deleterious effects of removing microglia and/or astrocytes (although, microglia-depleting CSF1R antagonists appear safe in clinical studies).^65^

It is important to note that there is no clear relationship between microglial morphology and their activation state, given that morphology depends on cellular adhesion and motility which differ between 2-dimensional cultures and the *in vivo* environment. ‘Activation’ is also a broad and poorly-defined term, but LPS modifies microglial morphology in these cultures, and this modification is altered by category 2 hits in particular. Similarly, there is no simple link between proliferation and activation, though microgliosis is frequently considered to indicate some form of activation under disease conditions.^66^ In this study, the classical inflammatory stimulus LPS induces proliferation as part of its broad effects on microglia. Many hits (particularly category 2) increased this proliferation, potentially by pushing microglia to alternative states that are both proliferative and neuroprotective, and which may have an altered morphology.

Details on all hit compounds can be found in Table 1, and their clustering analysis found in Figure 6A. We note that the BAY61 positive control reduced microglial numbers in the screen similar to what we found in our recent study focusing on this compound,^13^ but this was non-significant here likely due to fewer biological repeats and more stringent corrections for multiple comparisons.

### Screen hits

The top hits from the screen were assessed for relationships and overlap between their targets to prioritize these pathways for discussion here. For all targets of interest, the screen data was also checked for non-neuroprotective compounds which also had relevant activity, as their existence would reduce confidence in that target. Where identified, these are noted below as well.

Within the hit compound cluster 2, an unexpected enrichment for steroid signaling modulators was identified. Cholesterol is metabolized to steroid hormones within cells, including testosterone, estrogen, progesterone and cortisol, largely through cytochrome P450 (CYP) enzyme activities.^67^ Strikingly, out of the top 8 most protective hit compounds (all with >89% protection), 5 interfered with either CYP enzymes or steroid hormone receptors. Anastrozole (29) and bifonazole (67) together inhibited CYP3A4/17A1/19A1/51A1, though as 3 other compounds targeting CYP3A4 were not neuroprotective this target is of less interest. The remaining CYPs all have known links to neurodegeneration, with variants in each being associated with 2-fold or higher risk for AD or PD in certain populations.^68^^,^^69^^,^^70^^,^^71^^,^^72^ Notably, microglia have little to no expression of these genes, and the principal steroidogenic cells in the brain are instead astrocytes.^73^^,^^74^^,^^75^ Based on our data, steroids may regulate inflammatory neurodegeneration, potentially by limiting inflammation.

One important steroid signaling pathway downstream of CYP activity may be glucocorticoid signaling, as the strongest neuroprotective hit, triamcinolone (31), and the category 2 hit compound medroxyprogesterone acetate (114) both agonise glucocorticoid receptors (NR3C1). In the literature, there is strong evidence that glucocorticoid receptors in particular regulate macrophage functions, including microglia.^76^ Indeed, lack of glucocorticoid receptor in mice exacerbates inflammatory neurodegeneration in the context of stress, LPS, and Parkinson’s disease.^77^^,^^78^^,^^79^^,^^80^ The glucocorticoid receptor may also have neuron-intrinsic roles in neuronal health, as it can regulate gene expression and splicing in neurons, including that of the important neuronal homeostasis protein BDNF.^81^^,^^82^ Our data align with these established roles of the glucocorticoid receptor. This may explain some of the protective efficacy of CYP inhibitors, as these could in principle modify production of all steroids, including glucocorticoids.

Similarly, estrogen and progesterone signaling are particularly implicated in the LPS-induced neuronal loss. Anastrozole (29, category 2, see above) is an antagonist of CYP19A1 (also known as aromatase or estrogen synthetase), fulvestrant (28, category 2) is an estrogen receptor antagonist that also protects, and bifonazole (67, category 2) is an inhibitor of CYP17A1, required for synthesis of estrogen (and other steroids). Though not present in the ChEMBL data, bifonazole has also been reported to inhibit CYP19A1 with nanomolar potency.^83^ Interestingly, CYP19A1 antagonists have previously been found to reduce dementia risk in women with breast cancer,^84^ but CYP19A1 has reported neuroprotective roles as well.^85^ Collectively, our data support a role of estrogen in LPS-induced neuronal loss. Meanwhile, medroxyprogesterone acetate (114, category 2) is a progesterone analogue that activates the progesterone receptor and is used clinically to prevent ovulation and reduce menopause symptoms in women, and to reduce sex drive in men. Progesterone has various neuroprotective effects, including reducing neuroinflammation.^86^ Overall, our data suggests that progesterone protects and estrogen promotes LPS-induced neuronal loss, as this can be prevented by any of the above compounds.

Finally in relation to steroid signaling, compound 130 was 69% protective and agonises the liver X receptors-α/β (LXRs), which are activated by cholesterol derivatives and directly activate LXR-responsive genes.^87^ LXRs have established roles in microglia/macrophage regulation, but also astrocytes and oligodendrocytes.^88^ Notably, agonism inhibits NOS2 expression, NO production and proinflammatory cytokine release in LPS-treated microglia and astrocytes.^89^^,^^90^^,^^91^^,^^92^ *In vivo* LXR knock-out exacerbates autoimmunity in WT mice and amyloid-β pathology in APP/PS1 mice, while agonism ameliorates pathology in experimental autoimmune encephalitis and intracerebral haemorrhage.^89^^,^^90^^,^^91^^,^^93^ Overall, our hits indicate that steroid signaling and related pathways are important for inflammatory neurodegeneration.

Adrenoreceptor (ADR) modulators were also amongst the hit compounds. There are three subgroups of adrenoreceptors: α_1_ (G_q_-coupled), α_2_ (G_i_-coupled), and β (G_s_-coupled). Previous studies suggest that cerebellar neurons express high levels of α_2_-ADRs while microglia highly express β_2_-ADRs and low levels of α_1A_, with induction of α_2A_ expression after LPS treatment.^94^^,^^95^^,^^96^^,^^97^^,^^98^ The endogenous agonists noradrenaline and adrenaline (30, 109) were both ∼88% protective, which may reflect the known neuroprotective functions of β_2_-ADRs in microglia/macrophages.^99^ β_2_ agonism reduces Parkinson’s disease risk in human populations^100^^,^^101^ and can reduce microglial inflammation and phagocytosis both *in vitro* and *in vivo*.^102^^,^^103^^,^^104^^,^^105^^,^^106^^,^^107^^,^^108^^,^^109^ Interestingly, there were 3 ADR antagonists that protected neurons in our screen. However, two of these (doxazosin and alfuzosin, 111 and 112) are relatively specific α_1_-ADR inhibitors, so would not block endogenous neuroprotective activity from β-ADRs while instead inhibiting potentially proinflammatory α_1_-ADRs.^110^^,^^111^^,^^112^^,^^113^ The remaining ADR antagonist, labetalol (6), inhibits both α_1_-ADR and β-ADRs, which alongside its low potency may explain its lower protective efficacy (37%). Finally, some ADR modulators were not protective including the α_1_-and β-ADR agonist isoproterenol (83),^114^ which is compatible with β-ADRs protecting while α_1_-ADRs are detrimental. Amiodarone (108, α_2_-ADR inhibitor among other activities), dexmedetomidine (115, α_2_-ADR agonist) and flunarizine (73, weak α_2_-ADR inhibitor) also did not protect, indicating that α_2_-ADRs do not regulate LPS-induced neuronal loss.

A number of the hit compounds were anti-inflammatory, consistent with the inflammatory nature of the LPS-induced model, including resatorvid (214, TLR4 inhibitor), an SYK inhibitor (26), a NOS2 inhibitor (22), triamcinolone (31, glucocorticoid receptor agonist), a liver X receptor antagonist (130), OTS964 (152, TOPK/PBK and NEK1 inhibitor), and zafirlukast (105, cysteinyl leukotriene receptor 1/2 and MAPK14 inhibitor). CYSLTR1 antagonists have previously been found to be protective in rodent models of brain trauma, stroke, multiple sclerosis, Parkinson’s disease and AD.^115^ This increases confidence that hits from this screen may be protective in relevant brain pathologies.

Finally, MAPK pathway proteins were strongly represented among hit compound targets, including MAPK14 (p38α MAPK), MAPK11 (p38β MAPK), MAPK1, upstream kinases (MAP2K1/2/5) and the downstream kinase MAPKAPK2 (from compounds 105, 26, 165, 119, 96, and 210). MAPK signaling is diverse, but p38 MAPK signaling may be particularly relevant based on the higher protective effects observed for inhibitors of this MAPK class. Notably, p38 MAPK activity is increased in AD and may contribute to hyperphosphorylation of tau.^116^^,^^117^^,^^118^^,^^119^ More relevant to our cultures, p38 MAPKs are also pivotal in mediating proinflammatory microglial responses to tau and LPS, potentially via MAPKAPK2.^120^^,^^121^^,^^122^ Interestingly, β-ADR, steroid receptor and LXR agonism all exert some of their anti-inflammatory effects by diminishing microglial p38 MAPK activation.^90^^,^^107^^,^^123^ We note that erlotinib (101) inhibits MAP2K5 but was not protective, and nor was the p38α/β inhibitor VX-72 (121). However, there is sufficient evidence among the neuroprotective compounds to suggests that these MAPK proteins are of interest to control inflammatory neurodegeneration.

We have focused here on proteins/pathways that were strongly indicated to be of interest by multiple neuroprotective hits. Interestingly, many of these cellular targets have also been highlighted as novel druggable targets for AD treatment by the US National Institute on Aging-funded AMP AD and TREAT-AD consortiums, including CYP19A1, LXRs, p38 MAPKs, NR3C1, and SYK.^124^ However, there are many other interesting treatment effects in the data, and we encourage those interested to explore this further. Other proteins of interest include γ-aminobutyric acid receptors, heat shock protein 90, PDZ binding kinase, NIMA related kinase 1, and bromodomain-containing proteins.

### Screen methods

Our screen combined three relatively novel approaches: (1) use and automated characterization of mixed cultures, which enables complex physiology and pathology to be analyzed by high-content imaging, (2) modeling disease in a dish, enabling screening for compounds that prevent disease phenotypes (disease phenotypic screening), and (3) screening using an annotated library of compounds with known targets, potentially enabling identification of neuroprotective drugs, targets and physiological compounds.

Phenotypic screening with neurons has typically used monocultures and simple measurement of neuronal numbers or synaptic density to identify targets of interest within neuronal development/survival or neurodegeneration.^125^^,^^126^^,^^127^^,^^128^ There are some exceptions to this, such as a recent study proposing screening with neuron-glia mixed cultures.^30^ However, this study extended to testing 28 conditions using live/dead cell counts and manual analysis of glial morphology. If scaled up, this approach would still only capture a limited amount of data. Another study established an impressive mid-throughput approach for modeling AD with iPSC-derived mixed cultures, and used this to test 70 compounds (at multiple concentrations) for their ability to improve high-content measurements of dendrites, axons, synapses, or cell counts.^25^ This detailed analysis of neuronal features has both advantages and disadvantages versus measuring coarser phenotypes across all cell types in culture. It would certainly be interesting to combine this advanced model with the type of analysis presented here. Other studies have used cell type classification in neuron-glia coculture high-content assays using commercial platforms,^23^^,^^24^ but the lack of open-source methods means that similar assays are challenging or impossible to replicate for most labs. In a notable exception to the use of *in vitro* two-dimensional cultures, over 1,000 compounds were tested for their ability to protect neurons survival against ischemia in mouse brain slices.^129^ While impressive, this was undoubtedly an extremely laborious study given the tissue and resources required to culture thousands of brain slices and manually count and score surviving neurons (though this might now be automated). The same researchers reviewed brain slice models as a platform for drug discovery and conducted a similar screen for Huntington’s disease;^130^^,^^131^ however, the approach has not become established. We present a slightly less physiological but substantially more practical approach, such that this screen was conducted over a few months by a single researcher. In addition, multidimensional data beyond just neuronal counts is extracted here, and in an automated manner.

We identified that live-cell staining and imaging was preferable here as microglia were largely washed off by standard immunocytochemistry (ICC) protocols. This may be a peculiarity of these cerebellar neuron-glia cultures: cerebellar microglia are particularly round and non-ramified *in vivo*,^132^ and *in vitro* we and others find that they adopt a prominent and exposed position on the surface of cultures.^56^^,^^133^^,^^134^ Elsewhere, microglia are more clearly ramified and adherent, which likely renders them resilient to wash steps and ICC. In our case, we therefore continued with a live-cell staining approach which also proved practical and effective. We did not test other fixation protocols given that ICC was not essential to achieve the staining required for our intended assay, but these may improve the resilience of microglia to repeated wash steps if ICC is required.

On the image analysis, we confirm here that deep learning-based segmentation via Cellpose is powerful even for challenging segmentation tasks, outperforming other automated segmentation frameworks.^135^ Meanwhile, machine learning (ML) is also well-established for classification, having been used elsewhere to distinguish: unstained mammalian cell lines in coculture;^38^ hepatocytes and fibroblasts;^41^^,^^44^ endothelial cells and fibroblasts;^43^ nuclear morphologies within HeLa cells;^42^ and subcellular localization of proteins in yeast.^40^ ML-based methods can power highly reproducible image analysis while being more sensitive to subtle phenotypic changes than manual analysis.^31^ However, application within translational neuroscience remains limited. Combined with modern experimental tools, the groundwork now exists for easier use and analysis of complex neuron-glia mixed cultures at mid-to high-throughput. ML-based image analysis methods will likely be essential for this research.

### Limitations of the study

The methods presented here may be better applied to different mixed cultures depending on the experimental aims. For example, cerebellar cultures are only directly relevant to neurodegenerative diseases affecting this brain region, such as ataxia-telangiectasia, and cerebellar neurons and microglia are somewhat distinct within the brain.^132^^,^^136^ Cerebellar cultures have been extensively used due the high yields of relatively homogeneous cerebellar granule neurons that they produce (these neurons being the most abundant in the brain).^137^ However, the methods used here could in principle be extended to brain cells from regions of the brain that are more frequently the focus of neurodegenerative diseases such as the cortex or hippocampus. Alternative neurodegenerative stimuli may also be preferred depending on the experimental aims. Here we used LPS, which models neuroinflammation and has been directly implicated in neurodegenerative diseases including AD.^48^^,^^49^^,^^50^ Additionally, LPS has been widely used to recapitulate Parkinson’s-like pathology,^47^ and LPS treatment also alters microglial transcriptomes to resemble those from AD.^138^ However, to model a specific disease such as AD our approach would be improved through use instead of hippocampal neuron-glia cultures (for which we have validated our staining and analysis method here also) and/or a more physiological insult such as amyloid-β or homogenates from AD-afflicted brains.^25^^,^^125^

In this study we use primary cell cultures to model neurodegeneration. However, scalability is a pressing concern for primary cultures in screens, as terminally differentiated primary cells cannot be expanded like immortalized cells or stem cells. Here, we achieved sufficient cells through use of rats instead of mice and postnatal animals instead of prenatal animals. In addition, miniaturization to 384-well microplates nearly quadruples available wells compared to 96-well assays, and here we reliably generated over 1,500 wells of cells per preparation. For future work, it may be possible to pool the litters of multiple time-mated animals, and whole-brain or cortical culture preparations could produce significantly higher yields than the cerebellar preparations here.

We also noticed small variations in cell counts and proportions within and between culture plates and repeats in this study, as detailed in Figure S5. This correlated with the column-wise liquid handling used to plate cells during culture preparation and may be mitigated in the future by using plate-wise liquid handling to seed all cells simultaneously from a well-mixed stock, which is common in mid- and high-throughput cell culture. Though our treatment randomization was effective and appeared to remove any bias associated with this, at the very least this will have increased the technical variability of our results. Our quality control analyses of Figure S5 also highlighted repeat 2 of the screen to differ substantially from the other repeats with respect to cell counts and proportions, seemingly as a result of an overly high initial seeding density. Normalization of neuron and microglia counts was possible and effective, but the effect of altered cell numbers and proportions on complex biology may not be easily corrected by such an approach. We retained these data as repeat 2 cultures responded to LPS similar to other cultures, but future screens would benefit from more stringent control of seeding density using automated cell counting (which we found to be challenging for primary cell preparations with considerable amounts of debris) and alterations to liquid handling as above.

More fundamental limitations to the model presented here include the following: results with rodent cells may not successfully translate into humans due to species-specific biology; dissociation of cells from brain tissue may significantly alter the state of sensitive cells types like microglia;^139^ and the use of brain tissue from neonatal animals (as in this study) produces cells that are young relative to the aged cells of interest in most neurodegenerative disease. Additionally, here brain tissue was used irrespective of biological sex, producing a non-physiological mixed-sex culture. This may be particularly relevant to the steroid signaling hits identified in the screen, and it would be of interest to conduct similar assays in the future with cultures of male and female cells separately and compare the results. Next, the cultures used here were maintained in non-defined, serum-containing media that may affect baseline neuronal and microglial phenotypes, as has been found in other culture systems.^18^^,^^140^^,^^141^ It would be of benefit for future work to use modern protocols with defined media lacking serum, but this was not possible to optimize within the constraints of this project. Finally, we have not studied myelination in these cultures and whether this affects neurodegenerative assays or could be a useful phenotype to assess in future assays. However, the time span of the cultures used here is shorter than what is usually necessary to observe myelination *in vitro*, at least for hippocampal and cortical neurons.^142^^,^^143^^,^^144^

Looking forwards, it will become increasingly important to use more disease-relevant models. It is now feasible to conduct similar work using iPSC-derived cells with greater potential throughput, albeit greater expense. Individual cell types need to be separately differentiated and mixed carefully to generate self-sustaining cultures which reflect the *in vivo* milieu, and this approach has recently been used to produce a useful triculture model of AD.^25^ Indeed, iPSCs will be central to future translational biology, not least given their human origin and the possibility of patient-specific cellular assays. iPSC-derived neuron-glia cultures could be analyzed as reported here to maximize data extraction, and all live-cell stains used here are effective on human cells including IB_4_ and NeuO.^46^^,^^145^^,^^146^^,^^147^^,^^148^ The use of iPSC-derived cultures also avoids the disruptive tissue dissociation necessitated by primary cell cultures. However, iPSC-derived cells do not address the issue of the age of the cells as the pluripotent state reprograms cells to a young state; this limitation may be better addressed with transdifferentiated somatic cell models.^149^^,^^150^ Meanwhile, 3D organoid cultures are progressing such that their use in future screens may be considered.^2^^,^^4^^,^^5^^,^^6^^,^^151^ It is easy to imagine a similar high-content pipeline for organoids as has been achieved for whole zebrafish.^152^ For the moment, however, these cultures largely lie beyond current screening capabilities.

A primary output in this study was neuronal survival/loss in the face of a neurotoxic insult. Nonetheless, the abstracted approach of fully classifying neuron-glia mixed cultures is compatible with different phenotypic assays, such as screening for synaptic loss or changes to neuronal connectivity. This could increase the experimental value of such experiments; for example, analysis of microglial morphology would be relevant when screening for modifiers of synaptic density given microglial control of synapse dynamics. However, this would require co-staining with markers visualizing the phenotype of interest, which is limited by our standard staining (Hoechst, NeuO and IB_4_-AF594) to the one remaining channel on a 4-colour microscope, and ICC for NeuN would need to replace NeuO if fixation were required to visualize the desired phenotype. Integration of brightfield/phase-contrast information could make some of this staining redundant for accurate classification given the distinct morphologies of different cell types,^38^^,^^45^ and this would make our approach more flexible. There is also further information within the images from our approach that was not extracted here because the primary goal was to achieve robust quantification of cell types, and this is a caveat to our analysis of overall disease phenotypes. This includes data on neurite morphologies or the spatial relationships between cells, and these features could further distinguish hits from each other. Such measurements may be desirable depending on the experimental aims and could be explored further in the images from this study. More generalized machine learning frameworks such as contrastive learning on images themselves may be better suited to capturing all this complexity and analyzing comprehensive phenotypes, as opposed to designing algorithms that only measure specific features (such as cell counts).

The current approach is particularly limited with respect to astrocytes, for which we included no specific stain and therefore could not analyze morphology (though we separately confirmed that the nuclei being identified as astrocytic, based on their morphology, belong to GFAP^+^ cells). Though neurons and microglia are the most relevant cell types to the LPS-induced neurodegeneration assay here, further analysis of astrocytes may be important to understand some treatment effects and would ideally be included where possible. Many astrocyte markers can be visualized by ICC such as GFAP, and for live cells sulforhodamine 101 may be used as a preferential astrocyte marker (though there are potential toxicity and selectivity concerns with this stain).^153^^,^^154^

While we used live cell staining here, similar image analysis methods would be applicable to cultures from transgenic animals/cells with endogenous labeling, or cells which have been labeled by transfection. These approaches may provide more powerful staining of specific cellular targets and markers while avoiding the need for ICC. However, the use of endogenous labeling would limit the methods’ flexibility to new model systems and transfection can be challenging, particularly for primary cells and for microglia specifically.^155^ Transfection also frequently induces an inflammatory response in microglia due to their sensitivity to foreign DNA. Nonetheless, these options would be valuable and could be optimized in future work to add to this approach.

## STAR★Methods

### Key resources table

**Table:** 

REAGENT or RESOURCE	SOURCE	IDENTIFIER
**Antibodies**		

Mouse anti-NeuN, clone A60	Merck	MAB377; RRID: AB_2298772
Goat anti-Mouse IgG1 Cross-Adsorbed Secondary Antibody, Alexa Fluor 594	ThermoFisher Scientific	A-21125; RRID: AB_2535767

**Chemicals, peptides, and recombinant proteins**		

BAY61-3606 (hydrochloride)	Cayman Chemical	11423
Lipopolysaccharides from Salmonella enterica serotype typhimurium (LPS)	Merck	L6143

**Deposited data**		

Raw images	This paper	BIA: S-BIAD890
Image-level data (cell-type counts)	This paper	BIA: S-BIAD890

**Experimental models: Organisms/strains**		

Wild-type Wister rat (rattus norvegicus)	Charles River	Crl:WI

**Software and algorithms**		

ImageJ/FIJI	Schneider et al., 2012^57^; Schindelin et al., 2015^36^; Schindelin et al., 2012^61^	https://fiji.sc/ https://imagej.net/ij/
QuPath	Bankhead et al., 2017^33^	https://qupath.github.io/
CellProfiler	Stirling et al., 2021^34^	https://cellprofiler.org/
CellProfiler Analyst	Stirling et al., 2021^34^	https://cellprofileranalyst.org/
Cellpose	Stringer et al., 2021^34^	https://www.cellpose.org/
GraphPad Prism	GraphPad Software	https://www.graphpad.com/features
R	Chambers et al.	https://www.r-project.org/
R package: umap	Tomasz Konopka	https://github.com/tkonopka/umap
R package: viridis	Garnier et al., 20231^62^	https://sjmgarnier.github.io/viridis/
All analysis pipelines in this paper	This paper	https://github.com/timjyb/Birkle-et-al-2024-HCS

### Resource availability

#### Lead contact

Further information and requests for resources and reagents should be directed to and will be fulfilled by the lead contact, Guy Brown (gcb3@cam.ac.uk).

#### Materials availability

This study did not generate new unique reagents.

#### Data and code availability

• All images and image-level data generated in the screening study have been deposited at BioImage Archive and are publicly available as of the date of publication. The accession number is listed in the key resources table. Image-level data is also available in the supplemental information of this paper.
• CellProfiler pipelines and other image analysis code/approaches associated with this study are available on GitHub (https://github.com/timjyb/Birkle-et-al-2023-HTS). This is listed in the key resources table.
• Any additional information required to reanalyse the data reported in this paper is available from the lead contact upon request.

### Experimental model and study participant details

#### General cell culture

The following media and solutions were obtained from ThermoFisher: high-glucose DMEM (41965062), PBS (70011051), HBSS (14175095), and Versene (15040066). Penicillin/Streptomycin was from Sigma-Aldrich (P0781), as was poly-L-lysine (PLL; P4707) and LPS (L6143). Cell strainers were from Greiner Bio-One (542040). All cultures were maintained at 37°C in humidified 5% CO_2_ incubators and all media were supplemented with penicillin (100U/mL) and streptomycin (100μg/mL). All use of primary tissue complied with the UK Animals (Scientific Procedures) Act 1986 and was approved by the local ethical committee at Cambridge University. Cultures were prepared from all available animals regardless of sex as separating the study by sex would have severely limited throughput, which was a key goal for this work. This is further discussed in the Discussion.

#### Primary cerebellar neuron-glia cultures

Neuron-glia cultures were prepared as previously described.^156^ Briefly, P3-7 rat cerebella were dissected in ice-cold HBSS, finely diced, and incubated in Versene for 5–10 min at 37°C. Cerebella from both male and female pups were used and pooled together. Cells were then mechanically dissociated by trituration, transferred to warm media, pelleted, resuspended, and 40μm-strained. Live cells were counted using trypan blue. For low-throughput experiments, cultures were seeded at 100,000 live cells/well in 96-well plates (655180) coated with 0.01% PLL, in 100μL media. For high-content screening, cultures were seeded at 30,000 live cells/well in 384-well high-content imaging plates (6057302) coated with 0.01% PLL, in 30μL media. For 96-well plate cultures only, debris was shaken off manually after 24 h and the media replaced. Media consisted of high-glucose DMEM supplemented with 5% certified fetal bovine serum (FBS; Gibco; 10082147), 5% horse serum (Gibco; 26050088), 2mM L-glutamine, 13mM glucose, 5mM HEPES, and 20mM KCl.

#### Primary hippocampal neuron-glia cultures

Hippocampal neuron-glia cultures were generated using a published protocol^157^ with minor alterations. Specifically, cells were counted prior to plating to ensure consistent plating at an optimised 50,000 cells per well in 96-well plates (655180), and AraC treatment was omitted to ensure undisturbed glial populations in the cultures.

### Method details

#### NeuO versus NeuN comparison

96-well plate cerebellar neuron-glia cultures were treated at *DIV7* with the SYK inhibitor BAY61-3606 (11423) for 30 min prior to the addition of LPS at 100 ng/mL. At *DIV10*, cultures were stained for 1 h with topical addition of 200nM NeuroFluor NeuO^46^ (01801) and 1μg/mL Hoechst 33342 (62249). Without removal of staining media, cultures were imaged using the 10× objective on an EVOS M5000 epifluorescence microscope, with 4 images taken in consistent positions around each well. Cultures were then fixed with 4% paraformaldehyde for 15 min at room temperature, then washed once with 50mM NH_4_Cl-containing PBS and twice with standard PBS. Cells were permeabilised and blocked for 1 h with 0.1% TX-100 and 2% BSA in PBS, washed three times, and stained overnight at 4°C with 1:1000 mouse α-NeuN (MAB377) primary antibody in 1% BSA PBS. Cells were then washed three times, followed by 1 h room temperature incubation with 1:400 goat α-mouse AF594 (A21125) secondary antibody. After three more washes, cells were imaged using the EVOS M5000 microscope again. Fields of view were precisely aligned manually with the previously captured images of the NeuO staining, and illumination settings were used that provided comparable signal from α-NeuN immunostaining as was achieved with NeuO staining.

Analysis was performed using identical cell segmentation parameters on QuPath, then thresholding for positivity (either NeuO or NeuN) using thresholds set at the trough between negative and positive populations in the mean intensity histogram, to ensure that the detection threshold was comparable. To check for loss of microglia during fixation, microglia were manually counted from NeuO (pre-fixation) and NeuN (post-fixation) images from untreated control wells with the help of the phase-contrast channel, in which microglia have a distinct morphology.

#### Segmentation algorithm accuracy test

The accuracy of the different cell segmentation methods (as provided by ImageJ, QuPath, CellProfiler, and the CellProfiler plugin Cellpose) was performed manually. 3 cerebellar neuron-glia culture images were selected at random from 3 unrelated experiments, for 9 images total. The Hoechst 33342 channel images were subjected to background subtraction and then cropped, ready for analysis by each method. ImageJ (IJ) segmentation consisted of a Gaussian filter (1px radius) followed by Otsu auto-thresholding and a binary watershed operation; this was intended to be a basic segmentation approach. QuPath (QP) segmentation consisted of QuPath’s cell detection function using optimised parameters after exhaustive testing. CellProfiler (CP) segmentation consisted of CellProfiler’s IdentifyPrimaryObjects module using optimised parameters after exhaustive testing. Cellpose segmentation consisted of a RunCellpose module within CellProfiler using optimised parameters. Segmentation errors of the indicated types were counted manually with reference to the original Hoechst image. Code for each approach can be found on GitHub (https://github.com/timjyb/Birkle-et-al-2023-HTS).

#### Low-throughput imaging

The low-throughput cerebellar and hippocampal neuron-glia cultures used during assay development were stained at *DIV10* for 1 h at 37°C with topical addition of 200nM NeuroFluor NeuO (01801), 1μg/mL Hoechst 33342 (62249), and 1μg/mL IB_4_-AF594 (I21413). Without removal of staining media, cultures were imaged using the 10× objective on an EVOS M5000 epifluorescence microscope. Images were subject to background subtraction, Cellpose segmentation and feature measurement using the optimised CellProfiler pipeline (these can be found on GitHub at https://github.com/timjyb/Birkle-et-al-2023-HTS). CellProfiler Analyst was then used for manual training of classifiers.

#### Classifier validation and parameters

All available classifier models were tested to identify Random Trees and Support Vector Machines (SVM) as the most effective for our particular data. Other models available in CellProfiler Analyst included AdaBoost, GradientBoosting, LogisticRegression, LDA, KNeighbours, FastGentle Boosting, and Neural Networks, but these performed more poorly that Random Trees or SVM according to k-fold cross-validation (k = 5) with regards to both precision and recall. Classifiers trained for both cerebellar and hippocampal neuron-glia cultures were tested using the in-built k-fold evaluation available in CellProfiler Analyst (k = 5), including the classifier for the high-content screen. As the cerebellar neuron-glia culture classifier presented in Figure 3 was also to be applied to images from experiments beyond the training set, external validation was also performed by comparing classification on random cells from such experiments to manual annotations. Low-throughput cerebellar culture classification used a Support Vector Classifier, and hippocampal culture and high-content cerebellar culture classifications used Random Trees. The models and parameters used for each are available on GitHub (https://github.com/timjyb/Birkle-et-al-2023-HTS).

#### High-content screen compound filtering

Compounds were selected from the ADDI-LifeArc Annotated Library (as described in the Results),^158^ for which bioactivity data was extracted from ChEMBL31^159^ and combined with data from a limited selection of additional kinase assays. Based on this data, compounds were then filtered to achieve the compound set for the screen.

1. Compounds were excluded that had any of the following features:
  a 0 known single protein targets.
  b >9 known targets.
  c Topological polar surface area >120 (compounds unlikely to be cell permeant).
  d Best on-target potency >100nM by biochemical assays, or >1μM by cell-based assays.
2. 300 compounds were then randomly selected from the remaining list, and SYK inhibitors and a TLR4 inhibitor were included as positive controls for neuroprotection.
3 Finally, an iterative strategy was used to randomly remove compounds for which all their targets were already hit 3 or more times, as 2-fold overlap of compounds on a given target was considered optimal. This enabled random addition of some compounds adding new targets to the target set, or which added 2-fold overlap on existing targets. In this way, the compound set was reduced at random, while maintaining 2-fold overlap on as many targets as possible and without reducing the total number of unique targets hit by the compound set.

The final compound set consisted of 228 compounds targeting 439 unique targets. 328 protein targets were hit once by the compound set, 83 targets twice, and 29 targets were hit 3 to 6 times. One compound failed to dispense when preparing assay-ready plates, resulting in 227 compounds being tested in the final screen. New, up-to-date bioactivity data was used for final data analysis, resulting in minor alterations to annotated compound activities compared to those used for compound filtering. Approximate mechanisms of action (inhibition, agonism, binding, or unknown) were extracted from assay metadata for each compound in the compound set, and then manually verified for each compound-target interaction.

#### High-content screen compound organisation

Controls were DMSO (negative) and BAY61-3606 (positive). For each, there were 6 LPS- replicate wells per plate, and 6 LPS+ replicate wells per plate. This resulted in 252 wells total being used per plate, in a 18x14 layout.

Each compound was to be tested in the presence and absence of LPS, each in triplicate. As a result, six 384-well plates were necessary per repeat of the screen. We divided the compound set randomly in half and tested each half-set in three 384-well plates, with one LPS- and one LPS+ replicate for each compound present on each plate. This pairing of LPS- and LPS+ replicates was chosen to stop plate effects, the most likely source of experimental variation, from confounding the important LPS- vs. LPS+ comparison for each compound. Maintaining independent halves of the screen would also have allowed for screening of only one half-set, if made necessary by a low yield from a particular preparation of primary cells.

Within each plate, wells were divided evenly into LPS- and LPS+ wells in a chequerboard pattern, with LPS to be treated every other well. Within the LPS- area, one replicate for each compound was randomly assigned a location, and similarly for the LPS+ area. The 6 LPS- replicates and 6 LPS+ replicates for each control were also randomly assigned locations.

#### High-content screen plate generation

Having randomly distributed the LPS- and LPS+ replicates of each compound within each plate, assay-ready plates were generated using the Cherry Pick functionality of an Echo 520 instrument (Labcyte). 7.5nL of each compound or control was dispensed from 10mM DMSO stock plates into compound plates according to the randomised layout. Plates were centrifuged and stored at −20°C until use.

#### High-content screen treatments

On day of treatment, *DIV7*, assay-ready compound plates were warmed to room temperature and 6.25μL culture media was added per well. Plates were centrifuged to settle media and mix with the previously dispensed compound drops, resulting in 12μM final concentration for each compound/control. Using a custom program on a Viaflo384 (Integra) instrument, 3μL was transferred from each compound plate well to matching wells in a 384-well plate of *DIV7* neuron-glia cultures. After 30 min incubation, 3μL was then transferred to the same cultures from a ±LPS stock plate, with PBS or 1.2μg/mL LPS (in PBS, after 30 min water-bath sonication of 12μg/mL stock) arranged in the desired chequerboard pattern. This resulted in a final culture volume of 36μL, with 1μM of each treatment and ±100 ng/mL LPS per well.

#### High-content screen imaging

On *DIV10*, after 3 days of treatment, culture plates were sequentially taken for staining and endpoint imaging. For each plate, a fresh stain mix was prepared from identical stocks, distributed to one column of a 384-well intermediate plate, and then 4μL was added into each well via multichannel pipetting. This gave a final volume of 40μL per well. The final stain concentrations were 1μg/mL Hoechst 33342 (1 mg/mL stocks prepared fresh on each repeat; 62249), 1μg/mL Isolectin IB_4_-AF594 (I21413), 100nM NeuroFluor NeuO (01801), and 0.75μM DRAQ7 (DR71000). Plates were incubated at 37°C for 1 h and then, without removal of staining media, imaged at 37°C on an IN Cell Analyzer 6000 instrument (GE Healthcare), with 4 fields of view captured per well (covering approximately 80% of the well) and taking around 30 min per plate. As this was an endpoint assay, cells were only imaged once despite being ‘live’. All images are available on BioImage Archive.

#### High-content screen cell classification

The final CellProfiler pipeline used to process images and generate cell object measurements from the screen can be found on GitHub at (https://github.com/timjyb/Birkle-et-al-2023-HTS; Pipe1). Background subtraction was omitted owing to the uniformity of image illumination achieved on the high-content imager, which helped reduce computation time. The pipeline saves object images suitable for reloading into CellProfiler for further analysis, which avoids the need to rerun the computationally costly Cellpose segmentation again. Object outline images are also saved, which can be loaded into CPA alongside the original images for clear visualisation of objects (and mis-segmentation events) during classifier training. Object measurements were stored in an SQLite database, which was handled using the freely available DB Browser for SQLite software (https://sqlitebrowser.org/).

A randomly selected subset of the final object set from each repeat (all cells from all conditions) was then loaded into CPA (this reduced the tile fetch times while fetching objects for classification in CPA), and randomly selected objects from this set were classified manually into 7 classes, aiming for 400 objects each: Neuron, Microglia, Astrocyte, Other, Condensed, Necrotic or Debris. Neurons were visually identified by NeuO-positivity; microglia by IB_4_-positivity; astrocytes by their large nuclei with dim Hoechst staining, and lack of other positive staining; other by their small, neuron-sized nuclei with elevated Hoechst staining and lack of other positive staining; condensed by their very small size with intense Hoechst staining and lack of DRAQ7 staining; necrotic by their very small size and DRAQ7-positivity; and debris by the lack of substantial Hoechst staining. Once all four repeats were completed, object sets and training sets were combined, resulting in approximately 1,600 total objects annotated per class (11,294 in total) and spread evenly across repeats such that the final classifier would be robust to any variation in cells, staining, or image quality over the entire screen. With the random cell selection, the training set was also balanced between LPS- and LPS+ wells (5,692 versus 5,582, respectively). According to 5-fold cross-validation, the Random Trees Classifier within CPA performed the best, and this was used for final analysis.

#### High-content microglial morphology analysis

The final CellProfiler pipeline used to generate microglial shape measurements from the screen can be found on GitHub (https://github.com/timjyb/Birkle-et-al-2023-HTS; Pipe2). Object images (nuclei) from Pipe1 were reloaded into CellProfiler alongside the original images. Objects were then measured identically to in Pipe1 and classified using the final classifier model generated in CPA, with filtering to select only nuclei classified as ‘Microglia’. These nuclei were then used to seed masks based on the microglial IB_4_-AF594 staining, using an IdentifySecondaryObjects module. The shape of these microglial masks were measured, and the median value for each shape statistic, from all microglia per image, was used for downstream analysis.

### Quantification and statistical analysis

In all cases, N is used to refer to biological repeats, which in our study are independent preparations of cells from different litters of animals. Where summary statistics are presented, these are always mean ± standard deviation. All further statistical details can be found in the figure legends.

#### General data processing and statistics

Statistical analyses were performed using GraphPad Prism 9 for Windows (GraphPad Software, San Diego, CA, USA) and were as stated in figure legends. Neuron and microglial count data from the high-content screen were normalised prior to analysis given the clear (and expected) differences between LPS- and LPS+ counts for these cell types. For neuron counts, data from each repeat was rescaled between 0 and 1 using the median of LPS+ counts as the minimum (0) and the median of the LPS- counts as the maximum (1), in an adaptation of typical Min-Max normalisation. For microglia counts, the LPS- median was used to set the minimum and the LPS+ median used for the maximum instead, to reflect the directionality in the raw data (neurons decrease with LPS, microglia increase).

For quality control, principal component analysis (PCA) was used on the cell counts data from each plate/row/column/row (as stated) after standardisation. Where indicated, PC1 and PC2 were assessed for outliers by ROUT (Q = 1%).

#### Uniform manifold approximation projection

UMAP^160^ analysis was conducted on the CellProfiler-generated single-cell level object feature data for all cells across four randomly selected DMSO LPS- images (15,710 cells total), one image being taken from each biological repeat of the screen. 127 object feature dimensions were included (Table S1), all data was standardised prior to analysis, and irrelevant features were excluded in the same way as for cell classification (Table S1). Analysis was performed in R using the umap package (https://cran.r-project.org/web/packages/umap/index.html) with default hyperparameters: n_neighbours = 15, n_components = 2, metric = euclidean, n_epochs = 200, input = data, init = spectral, min_dist = 0.1, set_op_mix_ratio = 1, local_connectivity = 1, bandwidth = 1, alpha = 1, gamma = 1, negative_sample_rate = 5, a = NA, b = NA, spread = 1, random_state = NA, transform_state = NA, knn = NA, knn_repeats = 1, verbose = FALSE, umap_learn_args = NA). The random seed used for Figure 3G was 472890146.

#### Hierarchical clustering and heatmapping

For heatmapping compounds, data for each parameter (neurons, microglia, etc.) was first min-max normalised across both LPS- and LPS+ data together (i.e., data for each parameter was rescaled such that the minimum data point was set to zero and the maximum data point was set to one, where data points were the per-compound means of non-normalised data for that parameter). Data was then clustered and displaying using the heatmap() function in R, using Euclidean distance for the distance function and complete linkage clustering via hclust() (code available on GitHub at https://github.com/timjyb/Birkle-et-al-2023-HTS). For heatmapping neuroprotective hits only (and DMSO, for comparison), the normalised data was filtered to only include data for these compounds, and clustering/heatmapping was conducted in the same way as before.

#### Software

All CellProfiler pipelines and CellProfiler Analyst classifiers can be found on Github under the user timjyb. CellProfiler 4.2.4^34^ was built from source and the neuron-glia cultures analysis pipeline included a Cellpose 2.1.0 module.^39^ CellProfiler Analyst 3.0^35^ was used for cell classification. ImageJ 1.52–1.53^36^^,^^57^ via FIJI^161^ and QuPath 0.3.0^33^ were also used for comparative analyses. General data plotting was performed using GraphPad Prism 9 for Windows (GraphPad Software, San Diego, CA, USA). The R-generated heatmaps of Figures 6 and S9 used the viridis color palette package for R,^162^ and R version 3.6.3 was used. UMAP analysis used the umap package for R, version 0.2.7.0.

## Supplemental information

Document S1. Figures S1–S9 and Tables S1–S3 and S5–S7

Table S4. Identifiers and annotated activities for all screen compounds

Table S8. Image-level cell count and microglial morphology screen data

## Footnotes

Supplemental information can be found online at https://doi.org/10.1016/j.isci.2024.109454.
