# Supplementary material for: Disease phenotypic screening in neuron-glia cocultures identifies blockers of inflammatory neurodegeneration
Source: iScience. 2024 Mar 8;27(4):109454. doi: 10.1016/j.isci.2024.109454 (PMC10973195; doi:10.1016/j.isci.2024.109454)
Supplement: Document S1. Figures S1–S9 and Tables S1–S3 and S5–S7 [file mmc1.pdf]

iScience, Volume *27*

Supplemental information

Disease phenotypic screening in neuron-glia cocultures identifies blockers of inflammatory neurodegeneration

Timothy J.Y. Birkle, Henriette M.G. Willems, John Skidmore, and Guy C. Brown

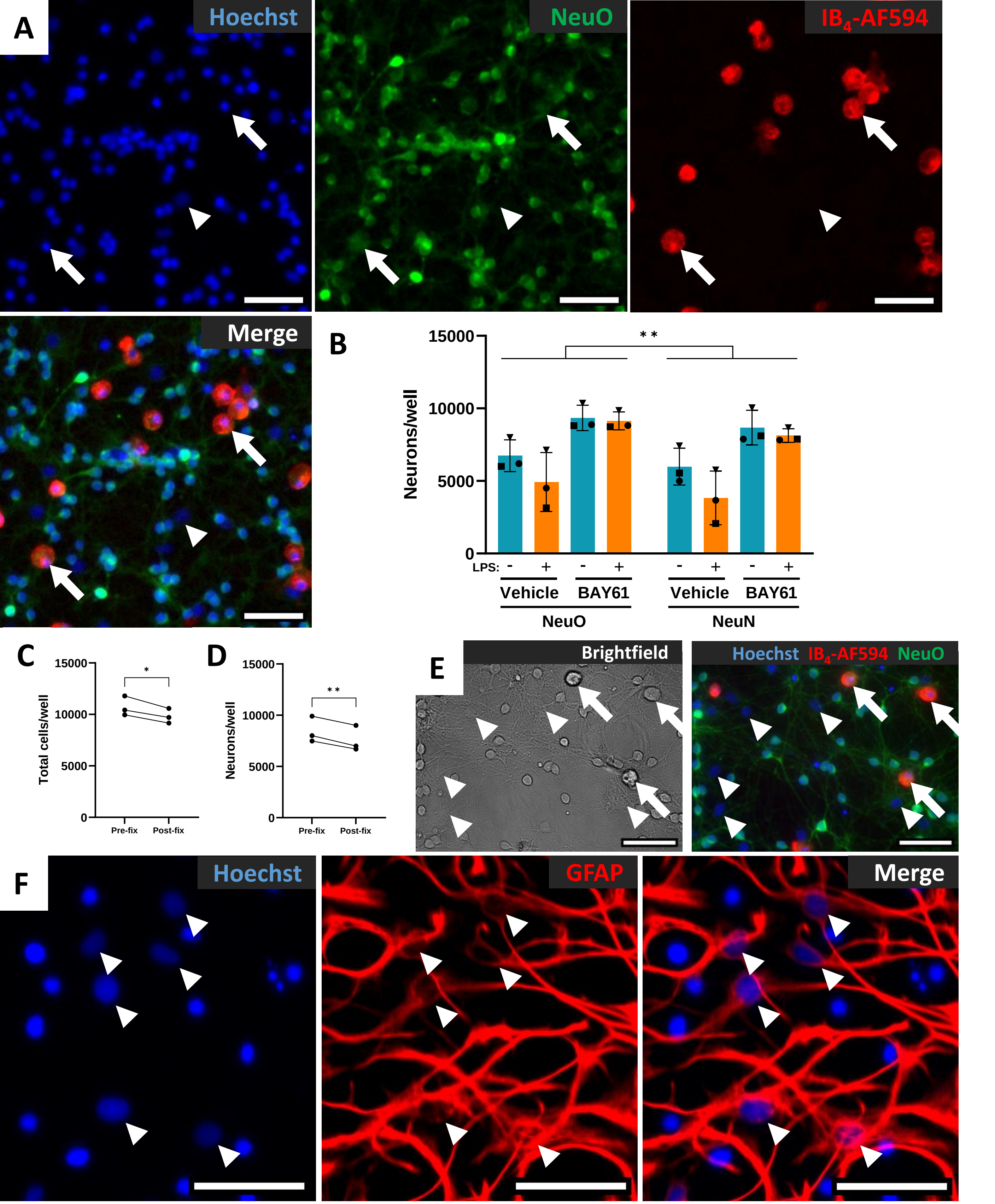

Figure S1. NeuO identifies neurons better than ICC for NeuN, which disturbs cells, related to Figure 1.

A: Representative 10x image (separate channels and merged; cropped) of neuron-glia cultures stained with Hoechst 33342 (nuclei), NeuO (live neurons), and IB_4-AF594 (microglia). Arrows indicate microglia (positively identified by IB_4 staining), arrowheads indicate astrocytes (positively identified by large nuclei with dimmer Hoechst staining). Scale bars = 50µm. **B:** Average neuronal counts per well (4 images) in cultures treated $\pm$ BAY61 (1µM) and $\pm$ LPS (100ng/mL) for 3 days (DIV10), quantified from matching fields-of-view with either pre-fix NeuO staining, or post-fix α-NeuN staining. RM 2-way ANOVA. Data are represented as mean $\pm$ SD. **C:** Average total cell counts per well (4 images) in untreated neuron-glia cultures, before and after fixation and ICC. Paired t-test. **D**: Average neuronal counts per well (4 images) in untreated neuron-glia cultures, before and after fixation and ICC. Paired t-test. **E:** Representative 40x image (brightfield and merged fluorescence channels) of cultures stained with Hoechst 33342 (nuclei), NeuO (live neurons), and IB_4-AF594 (microglia). Arrows indicate microglia (positively identified by IB_4 staining), arrowheads indicate astrocytes (positively identified by large nuclei with dimmer Hoechst staining). Scale bars = 50µm. **F:** Representative 20x image (separate channels and merged; cropped) of neuron-glia cultures stained with Hoechst 33342 (nuclei) and anti-GFAP immunocytochemistry (astrocytes). Arrowheads indicate astrocyte nuclei. Scale bars = 50µm. **All panels:** single datapoints are each the mean of 3 technical replicates, biological N = 3. * p<0.05, ** p<0.01.

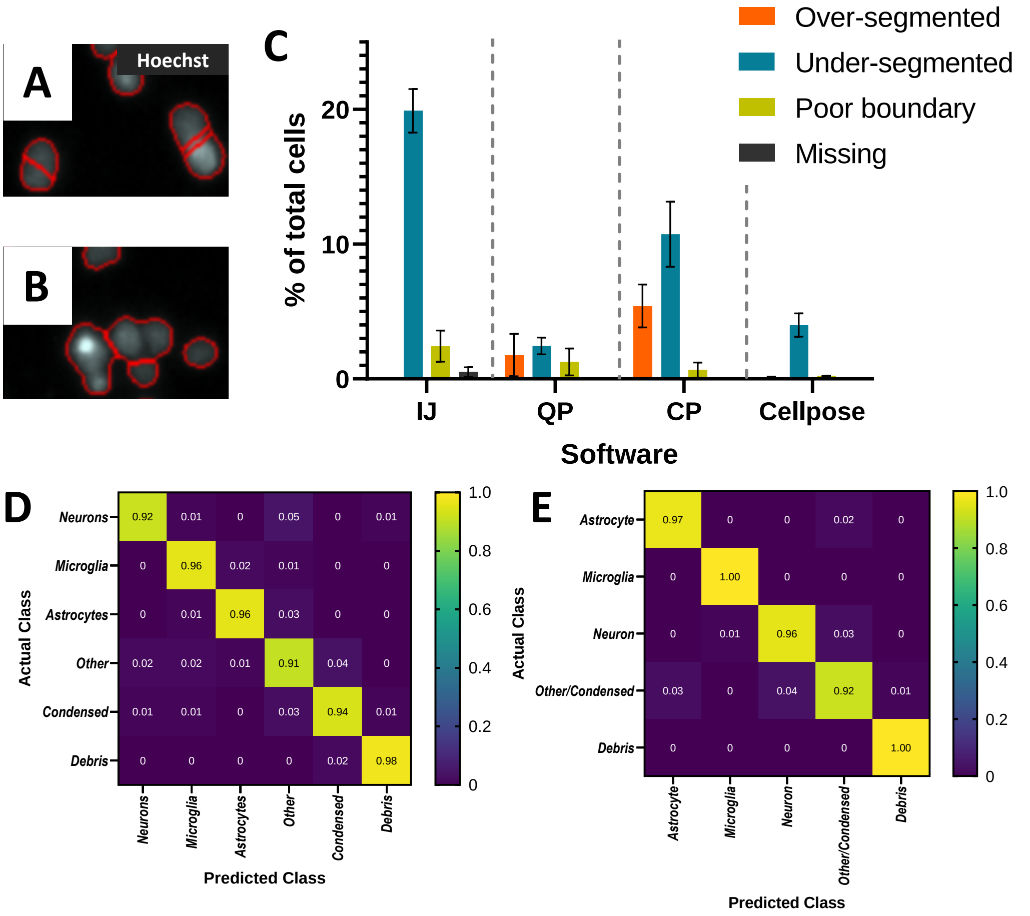

Figure S2. Machine learning-based analysis allows accurate, automated cell classification in neuron-glia cultures, related to Figure 2.

A: Example segmented Hoechst 33342 (DNA) image depicting over-segmentation error. **B:** Example segmented Hoechst 33342 (DNA) image depicting under-segmentation error. **C:** Percentage of total cells affected by segmentation errors when using optimised algorithms in ImageJ (IJ), QuPath (QP) and CellProfiler (CP), or using Cellpose. Data are represented as mean $\pm$ SD, N = 3. **D:** Cerebellar neuron-glia mixed culture classification accuracy - heatmap confusion matrix plotting proportion of manually annotated cells of each class (Actual) being predicted to be any given class (Predicted) by a classifier model during k-fold cross-validation (k=5). **E:** Hippocampal neuron-glia mixed culture classification accuracy - heatmap confusion matrix plotting proportion of manually annotated cells of each class (Actual) being predicted to be any given class (Predicted) by a classifier model during k-fold cross-validation (k=5).

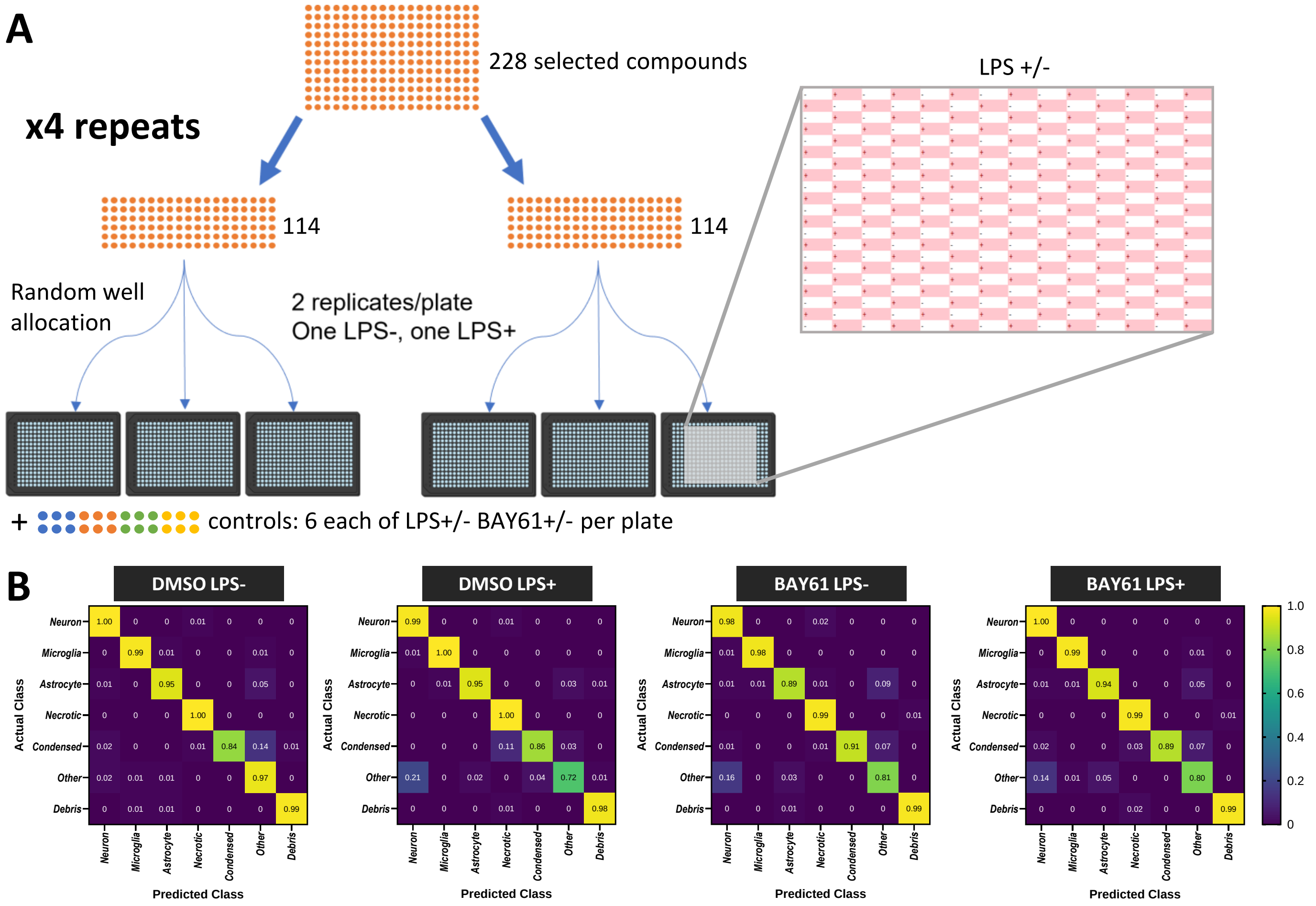

Figure S3. High-content screen design and classifier validation, related to Figure 3.

A: Schematic of the treatment distribution for the screen. Treatments were randomly assigned to 2 wells per plate within their half of the screen, 1 LPS-untreated and 1 LPS-treated. Controls were also randomly distributed. 1 treatment compound failed to dispense as intended, resulting in 227 compounds being tested in the final screen. **B:** Heatmap confusion matrices plotting the proportion of manually annotated cells of each class (Actual) being predicted to be any given class (Predicted) by the final trained classifier model from the screen, upon application to random images of each control condition (DMSO LPS-, DMSO LPS+, BAY61 LPS-, BAY61 LPS+) from all 4 screen repeats. 200 cells per class per condition were manually annotated for validation.

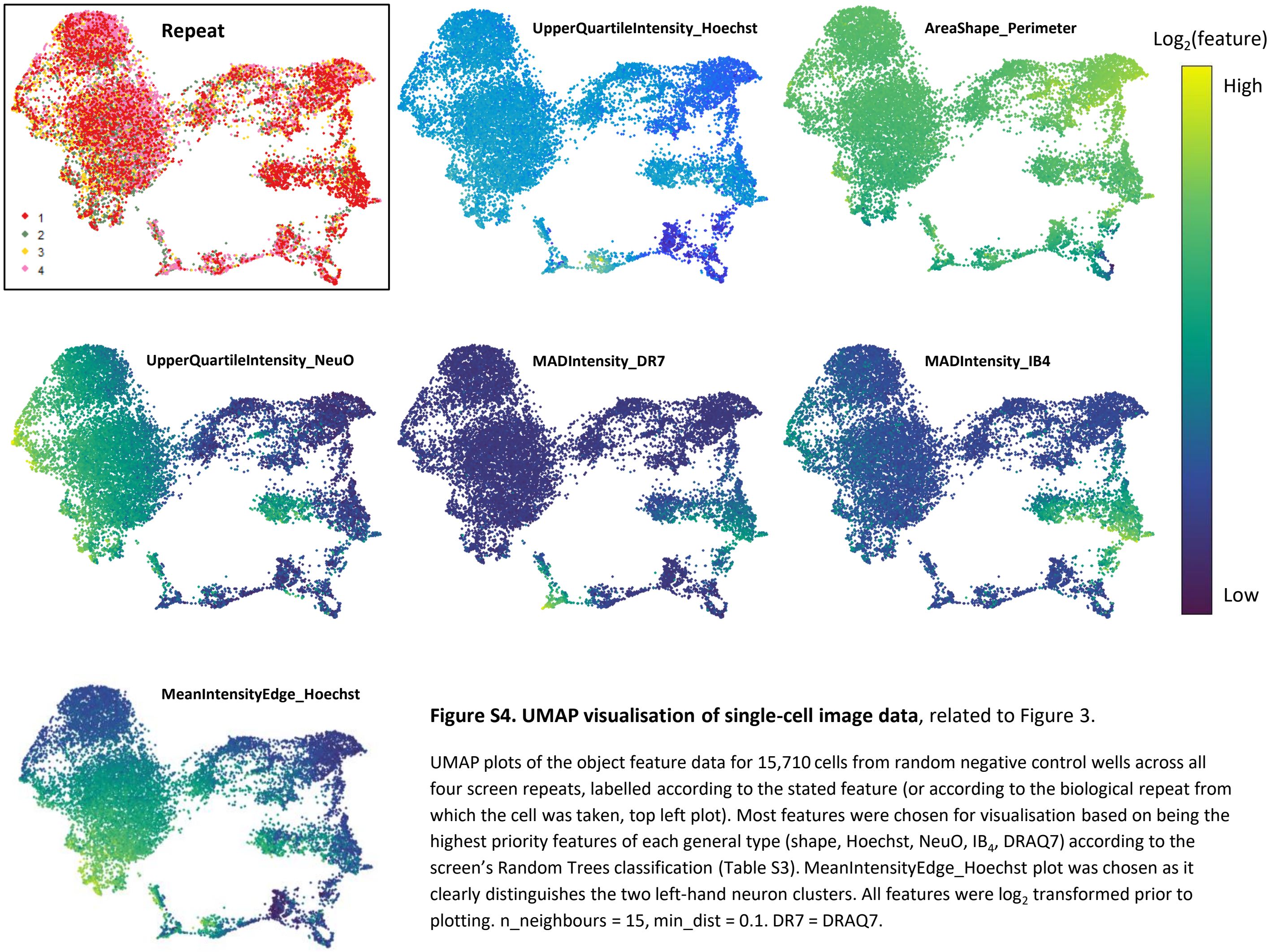

Figure S4. UMAP visualisation of single-cell image data, related to Figure 3.

UMAP plots of the object feature data for 15,710 cells from random negative control wells across all four screen repeats, labelled according to the stated feature (or according to the biological repeat from which the cell was taken, top left plot). Most features were chosen for visualisation based on being the highest priority features of each general type (shape, Hoechst, NeuO, IB_4, DRAQ7) according to the screen's Random Trees classification (Table S3). MeanIntensityEdge_Hoechst plot was chosen as it clearly distinguishes the two left-hand neuron clusters. All features were log_2 transformed prior to plotting. n_neighbours = 15, min_dist = 0.1. DR7 = DRAQ7.

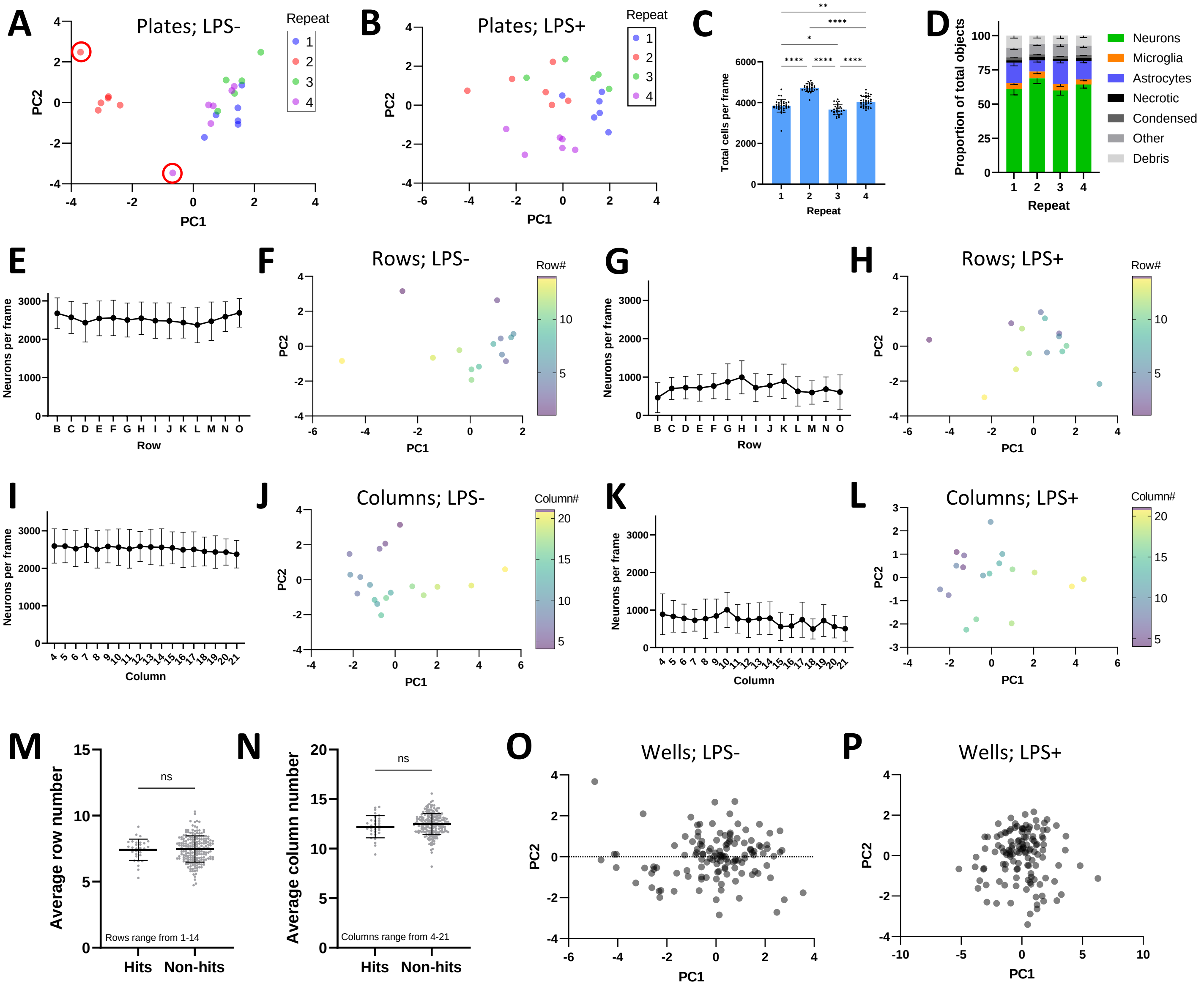

Figure S5. Screen data quality control, related to Figures 4 & 5.

A: Plot of PC1 and PC2 (combined 74.55% of variance) after PCA on the average count data of each cell type taken from the DMSO LPS- wells of each plate. Each datapoint represents the average of all control wells of one plate, and datapoints are colour-labelled according to the biological repeat which they were part of. Highlighted plates were identified as outliers by ROUT (Q = 1%). **B:** Same as (**A**), but for DMSO LPS+ wells instead (combined 66.33% of variance). No outliers detected by ROUT (Q = 1%). **C:** Average total cells per frame in DMSO LPS- wells from each biological repeat. Data are represented as mean ± SD, and individual datapoints represent the average total cells per frame for a single control well. One-way ANOVA with Tukey's *post-hoc* test. **D:** Average proportion of each cell type out of total cells for DMSO LPS- wells from each biological repeat. Data are represented as mean ± SD. **E:** Average neuron count per frame in each row of each 384-well plate, across all LPS- wells (controls and treatments combined). Data are represented as mean ± SD. **F:** Plot of PC1 and PC2 (combined 84.27% of variance) after PCA on the average count data of each cell type taken from all LPS- wells in each row across all plates. **G:** Same as (**E**), but for LPS+ wells instead. **H:** Same as (**F**), but for LPS+ wells instead (combined 83.34% of variance). **I:** Average neuron count per frame in each column of each 384-well plate, across all LPS- wells (controls and treatments combined). Data are represented as mean ± SD. **J:** Plot of PC1 and PC2 (combined 82.33% of variance) after PCA on the average count data of each cell type taken from all LPS- wells in each column across all plates. **K:** Same as (**I**), but for LPS+ wells instead. **L:** Same as (**J**), but for LPS+ wells instead (combined 71.34% of variance). **M:** Average row location (row B = 1, row 0 = 14) for each treatment over the course of all screen repeats, comparing treatment which were identified as neuroprotective hits versus non-hits. Unpaired t-test. **N:** Average column location for each treatment over the course of all screen repeats, comparing treatment which were identified as neuroprotective hits versus non-hits. Unpaired t-test. **O:** Plot of PC1 and PC2 (combined 54.08% of variance) after PCA on the average count data of each cell type taken from each well (LPS- only) across all plates. **P:** Plot of PC1 and PC2 (combined 66.93% of variance) after PCA on the average count data of each cell type taken from each well (LPS+ only) across all plates. **All panels:** * p<0.05, ** p<0.01, **** p<0.0001.

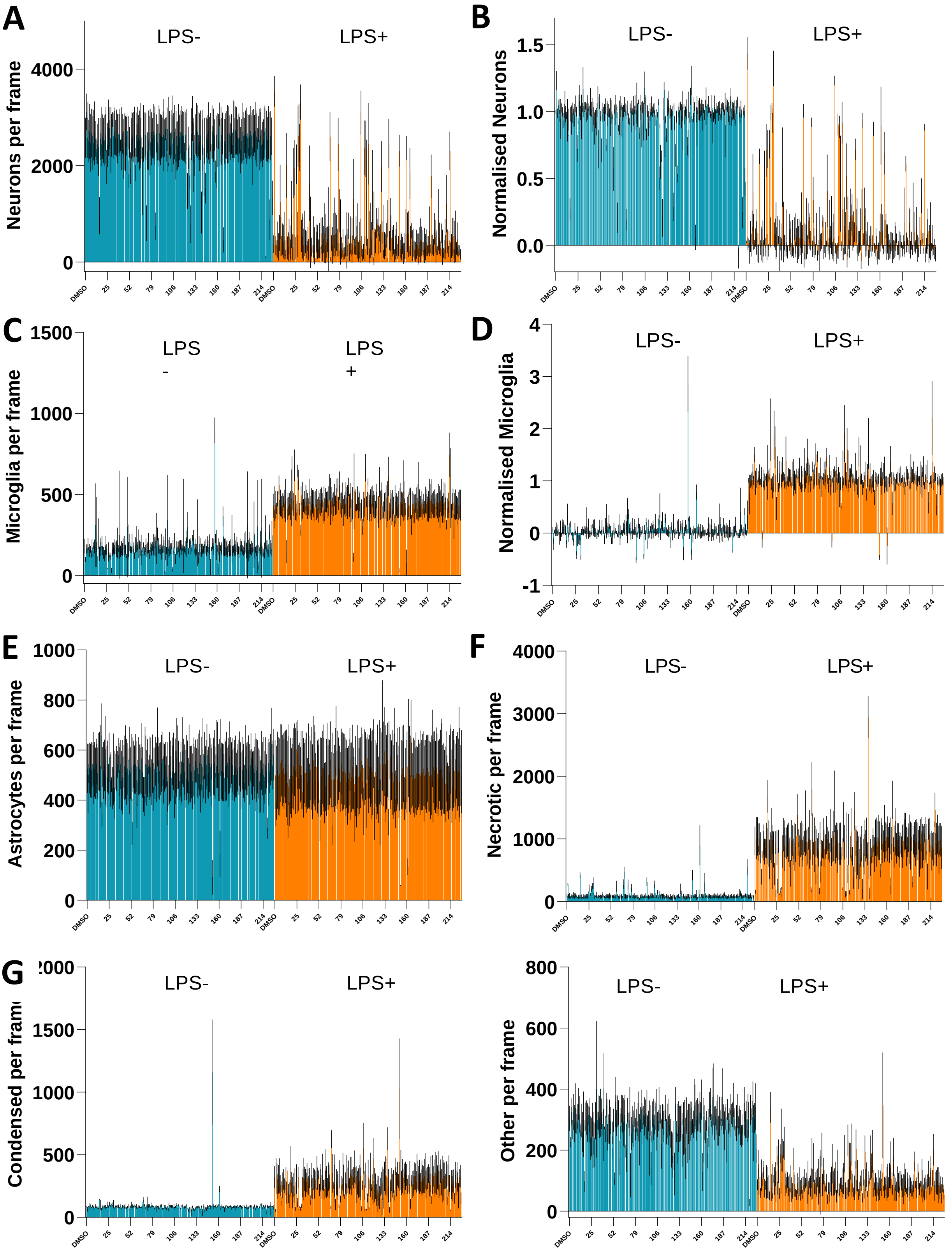

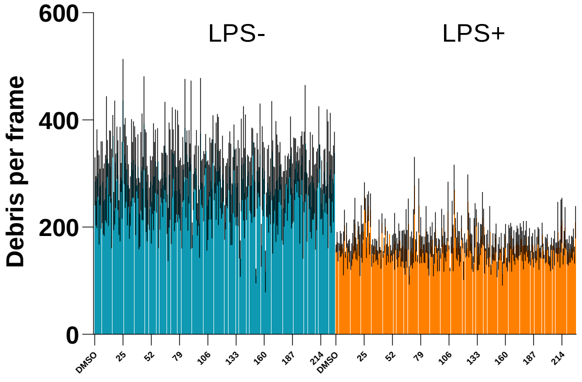

Figure S6. Raw data from screening 227 compounds for effects in the absence and presence of LPS, related to Figures 4-6.

A, B: Raw (**A**) and normalised (**B**) cell count data for Neurons in neuron-glia cultures treated ± LPS (100ng/mL) and ± each compound in the screen (1µM) for 3 days (*DIV10*). **C, D:** Raw (**C**) and normalised (**D**) cell count data for Microglia in neuron-glia cultures treated ± LPS (100ng/mL) and ± each compound in the screen (1µM) for 3 days (*DIV10*). **E-I:** Raw cell count data for each other class – Astrocytes, Necrotic, Condensed, Other, and Debris respectively – in neuron-glia cultures treated ± LPS (100ng/mL) and ± each compound in the screen (1µM) for 3 days (*DIV10*). **All panels:** Ordered according to arbitrary compound ID. Data are represented as mean ± SD, N = 4 biological repeats.

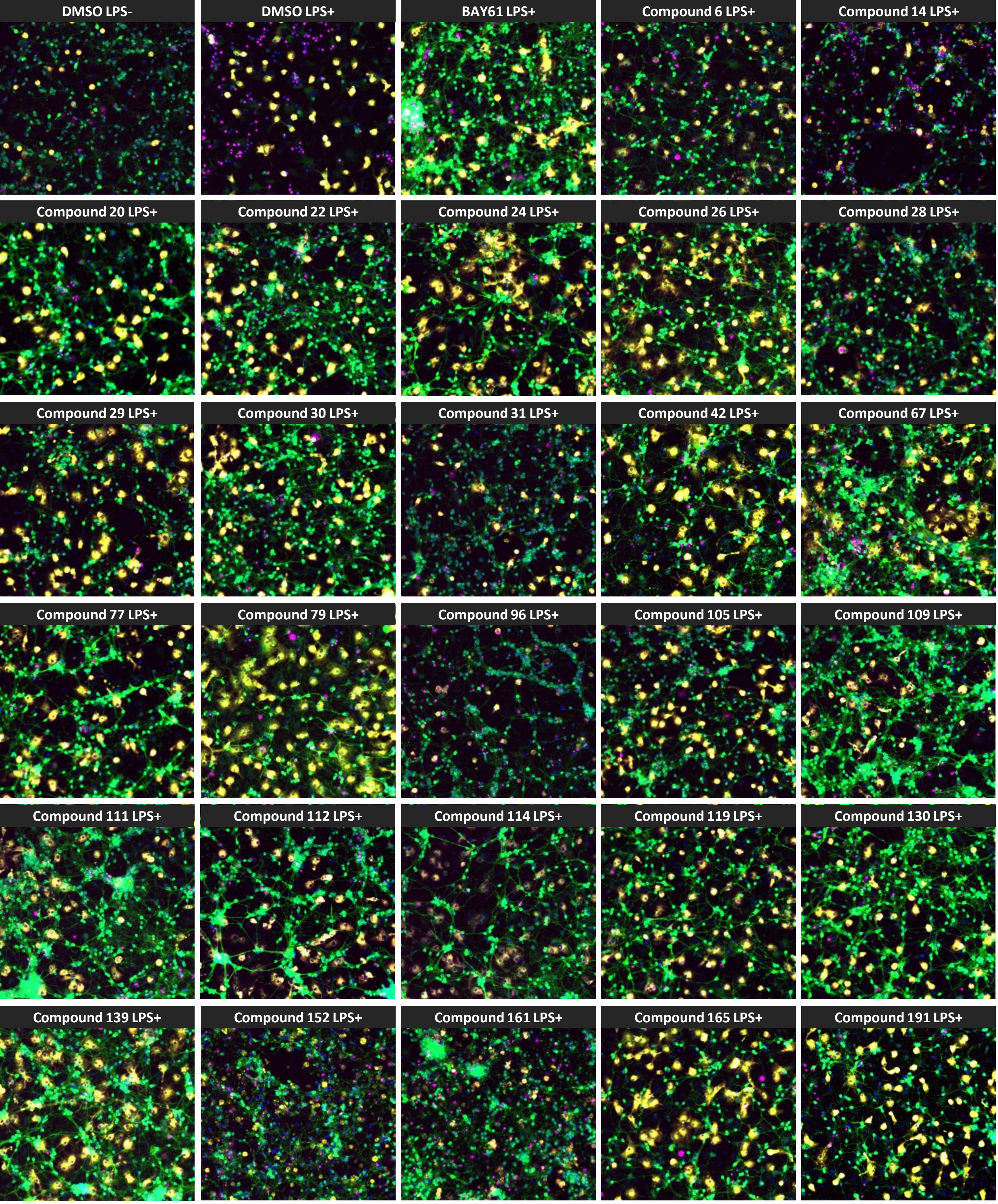

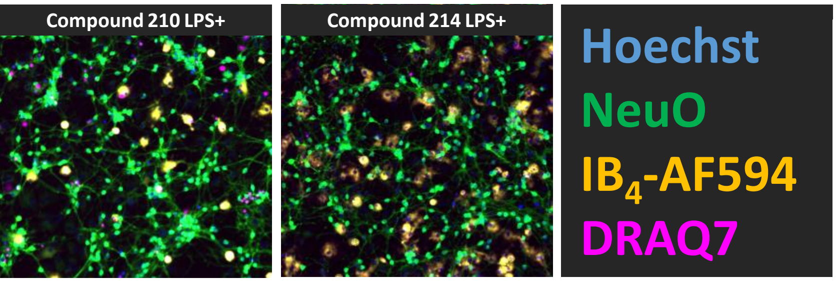

Figure S7. Images of cultures treated with all neuroprotective hit compounds in the presence of LPS, related to Figures 4-6.

Randomly selected images of cultures treated with all hit compounds in the presence of LPS, with DMSO LPS- and DMSO LPS+ images for comparison, after staining with Hoechst 33342 (nuclei), NeuO (live neurons), IB_4-AF594 (microglia), and DRAQ7 (necrotic cells). Image side lengths = 525µm.

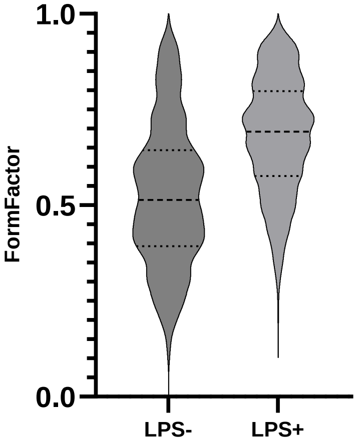

Figure S8. Diversity in microglial FormFactor, related to Figure 5.

Violin plot of the FormFactor (circularity) distribution of individual microglia from random DMSO-treated LPS- and LPS+ cultures across all four screen repeats. N(LPS-) = 1,050; N(LPS+) = 1,694.

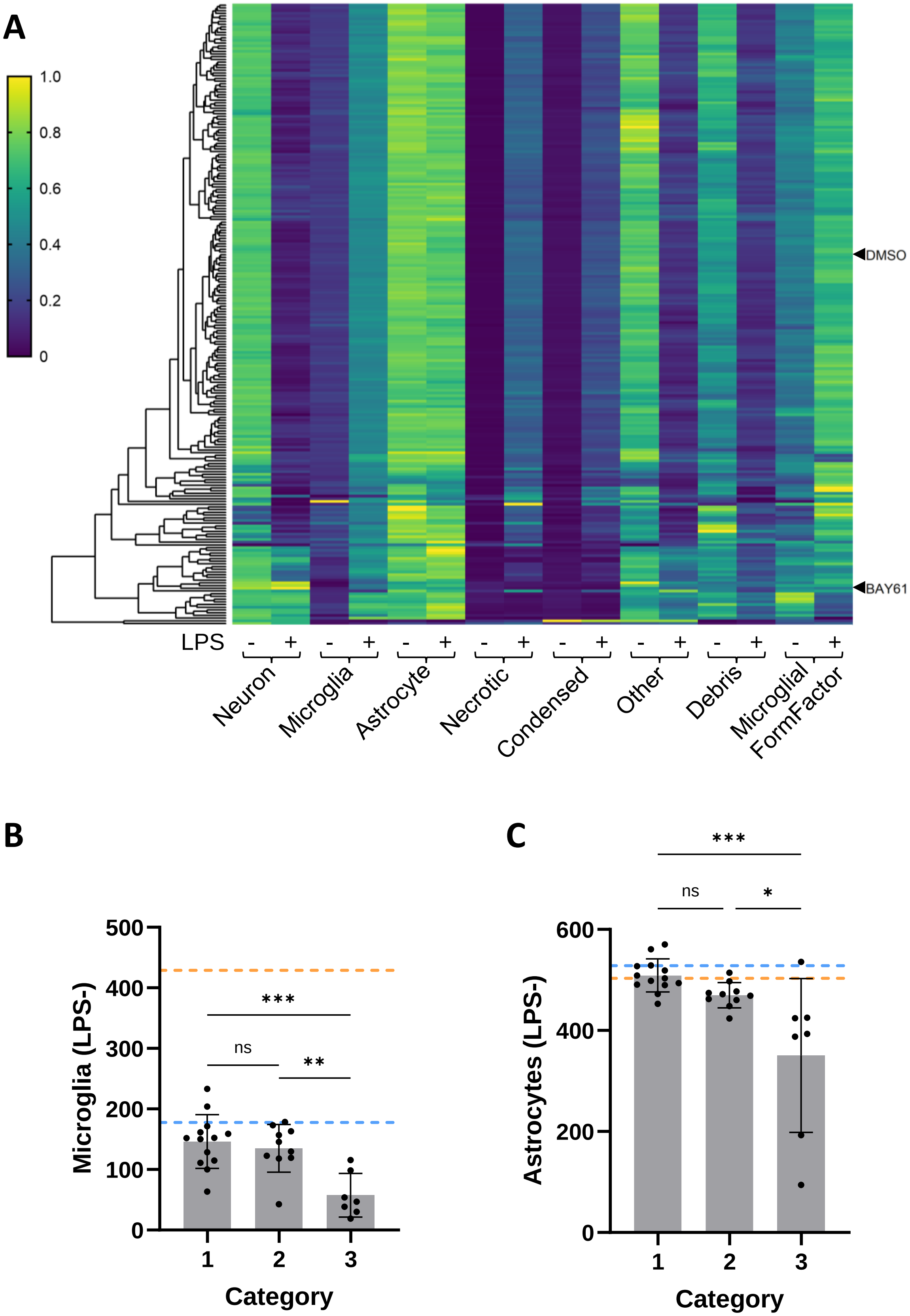

Figure S9. Full clustered heatmap analysis, related to Figure 6.

A: Clustered heatmap of all compounds (top to bottom) and all data (counts by each cell type and microglial morphology in the absence and presence of LPS). E.g. "Neuron –" and "Neuron +" columns display mean neuron counts in the absence and presence of LPS respectively. Each data type was min-max normalised between 0 and 1 based on min and max values across both LPS-treated and LPS-untreated conditions, to ensure comparability. **B:** Average microglial counts in the absence of LPS for hit compounds of each category. Each datapoint is the average count for one hit compound over all screen repeats. Categories 1, 2, and 3 included 13, 10, and 7 hit compounds respectively. One-way ANOVA with Tukey's *post hoc* test. Blue and orange dashed lines indicate the average microglial count in LPS-untreated and LPS-treated DMSO wells respectively. **C:** Average astrocyte counts in the absence of LPS for hit compounds of each category. Each datapoint is the average count for one hit compound over all screen repeats. Categories 1, 2, and 3 included 13, 10, and 7 hit compounds respectively. One-way ANOVA with Tukey's *post hoc* test. Blue and orange dashed lines indicate the average astrocyte count in LPS-untreated and LPS-treated DMSO wells respectively. * $p<0.05$, ** $p<0.01$, *** $p<0.001$.

	Feature	Excluded from classifier?	Reason
1	AreaShape_Area		
2	AreaShape_BoundingBoxArea		
3	AreaShape_BoundingBoxMaximum_X	x	Location data irrelevant
4	AreaShape_BoundingBoxMaximum_Y	x	Location data irrelevant
5	AreaShape_BoundingBoxMinimum_X	x	Location data irrelevant
6	AreaShape_BoundingBoxMinimum_Y	x	Location data irrelevant
7	AreaShape_Center_X	x	Location data irrelevant
8	AreaShape_Center_Y	x	Location data irrelevant
9	AreaShape_Compactness		
10	AreaShape_ConvexArea		
11	AreaShape_Eccentricity		
12	AreaShape_EquivalentDiameter		
13	AreaShape_EulerNumber	x	0 variance feature (all objects have EulerNumber = 1)
14	AreaShape_Extent		
15	AreaShape_FormFactor		
16	AreaShape_MajorAxisLength		
17	AreaShape_MaxFeretDiameter		
18	AreaShape_MaximumRadius		
19	AreaShape_MeanRadius		
20	AreaShape_MedianRadius		
21	AreaShape_MinFeretDiameter		
22	AreaShape_MinorAxisLength		
23	AreaShape_Orientation		
24	AreaShape_Perimeter		
25	AreaShape_Solidity		
26	Intensity_IntegratedIntensityEdge_DR7		
27	Intensity_IntegratedIntensityEdge_Hoechst		
28	Intensity_IntegratedIntensityEdge_IB4		
29	Intensity_IntegratedIntensityEdge_NeuO		
30	Intensity_IntegratedIntensity_DR7		
31	Intensity_IntegratedIntensity_Hoechst		
32	Intensity_IntegratedIntensity_IB4		
33	Intensity_IntegratedIntensity_NeuO		
34	Intensity_LowerQuartileIntensity_DR7		
35	Intensity_LowerQuartileIntensity_Hoechst		
36	Intensity_LowerQuartileIntensity_IB4		
37	Intensity_LowerQuartileIntensity_NeuO		
38	Intensity_MADIntensity_DR7		
39	Intensity_MADIntensity_Hoechst		
40	Intensity_MADIntensity_IB4		
41	Intensity_MADIntensity_NeuO		
42	Intensity_MassDisplacement_DR7		
43	Intensity_MassDisplacement_Hoechst		
44	Intensity_MassDisplacement_IB4		
45	Intensity_MassDisplacement_NeuO		
46	Intensity_MaxIntensityEdge_DR7		
47	Intensity_MaxIntensityEdge_Hoechst		
48	Intensity_MaxIntensityEdge_IB4		
49	Intensity_MaxIntensityEdge_NeuO		
50	Intensity_MaxIntensity_DR7		
51	Intensity_MaxIntensity_Hoechst		
52	Intensity_MaxIntensity_IB4		
53	Intensity_MaxIntensity_NeuO		
54	Intensity_MeanIntensityEdge_DR7		
55	Intensity_MeanIntensityEdge_Hoechst		
56	Intensity_MeanIntensityEdge_IB4		
57	Intensity_MeanIntensityEdge_NeuO		
58	Intensity_MeanIntensity_DR7		
59	Intensity_MeanIntensity_Hoechst		
60	Intensity_MeanIntensity_IB4		
61	Intensity_MeanIntensity_NeuO		
62	Intensity_MedianIntensity_DR7		
63	Intensity_MedianIntensity_Hoechst		
64	Intensity_MedianIntensity_IB4		
65	Intensity_MedianIntensity_NeuO		
66	Intensity_MinIntensityEdge_DR7		
67	Intensity_MinIntensityEdge_Hoechst		
68	Intensity_MinIntensityEdge_IB4		
69	Intensity_MinIntensityEdge_NeuO		
70	Intensity_MinIntensity_DR7		
71	Intensity_MinIntensity_Hoechst		
72	Intensity_MinIntensity_IB4		
73	Intensity_MinIntensity_NeuO		
74	Intensity_StdIntensityEdge_DR7		
75	Intensity_StdIntensityEdge_Hoechst		
76	Intensity_StdIntensityEdge_IB4		
77	Intensity_StdIntensityEdge_NeuO		
78	Intensity_StdIntensity_DR7		
79	Intensity_StdIntensity_Hoechst		
80	Intensity_StdIntensity_IB4		
81	Intensity_StdIntensity_NeuO		
82	Intensity_UpperQuartileIntensity_DR7		
83	Intensity_UpperQuartileIntensity_Hoechst		
84	Intensity_UpperQuartileIntensity_IB4		
85	Intensity_UpperQuartileIntensity_NeuO		
86	Location_CenterMassIntensity_X_DR7	x	Location data irrelevant
87	Location_CenterMassIntensity_X_Hoechst	x	Location data irrelevant
88	Location_CenterMassIntensity_X_IB4	x	Location data irrelevant
89	Location_CenterMassIntensity_X_NeuO	x	Location data irrelevant
90	Location_CenterMassIntensity_Y_DR7	x	Location data irrelevant
91	Location_CenterMassIntensity_Y_Hoechst	x	Location data irrelevant
92	Location_CenterMassIntensity_Y_IB4	x	Location data irrelevant
93	Location_CenterMassIntensity_Y_NeuO	x	Location data irrelevant
94	Location_CenterMassIntensity_Z_DR7	x	Location data irrelevant
95	Location_CenterMassIntensity_Z_Hoechst	x	Location data irrelevant
96	Location_CenterMassIntensity_Z_IB4	x	Location data irrelevant
97	Location_CenterMassIntensity_Z_NeuO	x	Location data irrelevant
98	Location_Center_X	x	Location data irrelevant
99	Location_Center_Y	x	Location data irrelevant
100	Location_Center_Z	x	Location data irrelevant
101	Location_MaxIntensity_X_DR7	x	Location data irrelevant
102	Location_MaxIntensity_X_Hoechst	x	Location data irrelevant
103	Location_MaxIntensity_X_IB4	x	Location data irrelevant
104	Location_MaxIntensity_X_NeuO	x	Location data irrelevant
105	Location_MaxIntensity_Y_DR7	x	Location data irrelevant
106	Location_MaxIntensity_Y_Hoechst	x	Location data irrelevant
107	Location_MaxIntensity_Y_IB4	x	Location data irrelevant
108	Location_MaxIntensity_Y_NeuO	x	Location data irrelevant
109	Location_MaxIntensity_Z_DR7	x	Location data irrelevant
110	Location_MaxIntensity_Z_Hoechst	x	Location data irrelevant
111	Location_MaxIntensity_Z_IB4	x	Location data irrelevant
112	Location_MaxIntensity_Z_NeuO	x	Location data irrelevant
113	Parent_ShrunkenNuclei	x	Parent data irrelevant
114	RadialDistribution_FracAtD_DR7_1of4		
115	RadialDistribution_FracAtD_DR7_2of4		
116	RadialDistribution_FracAtD_DR7_3of4		
117	RadialDistribution_FracAtD_DR7_4of4		
118	RadialDistribution_FracAtD_Hoechst_1of4		
119	RadialDistribution_FracAtD_Hoechst_2of4		
120	RadialDistribution_FracAtD_Hoechst_3of4		
121	RadialDistribution_FracAtD_Hoechst_4of4		
122	RadialDistribution_FracAtD_IB4_1of4		
123	RadialDistribution_FracAtD_IB4_2of4		
124	RadialDistribution_FracAtD_IB4_3of4		
125	RadialDistribution_FracAtD_IB4_4of4		
126	RadialDistribution_FracAtD_NeuO_1of4		
127	RadialDistribution_FracAtD_NeuO_2of4		
128	RadialDistribution_FracAtD_NeuO_3of4		
129	RadialDistribution_FracAtD_NeuO_4of4		
130	RadialDistribution_MeanFrac_DR7_1of4		
131	RadialDistribution_MeanFrac_DR7_2of4		
132	RadialDistribution_MeanFrac_DR7_3of4		
133	RadialDistribution_MeanFrac_DR7_4of4		
134	RadialDistribution_MeanFrac_Hoechst_1of4		
135	RadialDistribution_MeanFrac_Hoechst_2of4		
136	RadialDistribution_MeanFrac_Hoechst_3of4		
137	RadialDistribution_MeanFrac_Hoechst_4of4		
138	RadialDistribution_MeanFrac_IB4_1of4		
139	RadialDistribution_MeanFrac_IB4_2of4		
140	RadialDistribution_MeanFrac_IB4_3of4		
141	RadialDistribution_MeanFrac_IB4_4of4		
142	RadialDistribution_MeanFrac_NeuO_1of4		
143	RadialDistribution_MeanFrac_NeuO_2of4		
144	RadialDistribution_MeanFrac_NeuO_3of4		
145	RadialDistribution_MeanFrac_NeuO_4of4		
146	RadialDistribution_RadialCV_DR7_1of4		
147	RadialDistribution_RadialCV_DR7_2of4		
148	RadialDistribution_RadialCV_DR7_3of4		
149	RadialDistribution_RadialCV_DR7_4of4		
150	RadialDistribution_RadialCV_Hoechst_1of4		
151	RadialDistribution_RadialCV_Hoechst_2of4		
152	RadialDistribution_RadialCV_Hoechst_3of4		
153	RadialDistribution_RadialCV_Hoechst_4of4		
154	RadialDistribution_RadialCV_IB4_1of4		
155	RadialDistribution_RadialCV_IB4_2of4		
156	RadialDistribution_RadialCV_IB4_3of4		
157	RadialDistribution_RadialCV_IB4_4of4		
158	RadialDistribution_RadialCV_NeuO_1of4		
159	RadialDistribution_RadialCV_NeuO_2of4		
160	RadialDistribution_RadialCV_NeuO_3of4		
161	RadialDistribution_RadialCV_NeuO_4of4		

Table S1. All object features generated by the CellProfiler pipeline, related to Figure 2.
DR7 = DRAQ7.

Rank	Feature
1	Intensity_StdIntensity_Hs_Rescaled
2	Intensity_MaxIntensity_Hs_Rescaled
3	Intensity_IntegratedIntensity_Hs_Rescaled
4	AreaShape_Perimeter
5	Intensity_UpperQuartileIntensity_Hs_Rescaled
6	AreaShape_MajorAxisLength
7	AreaShape_MaxFeretDiameter
8	AreaShape_BoundingBoxArea
9	Intensity_IntegratedIntensity_IB4_Rescaled
10	Intensity_UpperQuartileIntensity_NeuO_Rescaled
11	Intensity_MADIntensity_Hs_Rescaled
12	Intensity_MeanIntensity_IB4_Rescaled
13	Intensity_UpperQuartileIntensity_IB4_Rescaled
14	Intensity_MADIntensity_NeuO_Rescaled
15	AreaShape_Area
16	AreaShape_EquivalentDiameter
17	AreaShape_ConvexArea
18	Intensity_MedianIntensity_IB4_Rescaled
19	Intensity_IntegratedIntensity_NeuO_Rescaled
20	Intensity_MaxIntensityEdge_IB4_Rescaled

Table S2. Top 20 features prioritised during Random Trees classification of cells during assay development, related to Figure 2.
Hs = Hoechst.

Rank	Feature
1	Intensity_UpperQuartileIntensity_Hoechst
2	Intensity_IntegratedIntensity_Hoechst
3	AreaShape_Perimeter
4	Intensity_MaxIntensity_Hoechst
5	Intensity_UpperQuartileIntensity_NeuO
6	Intensity_MADIntensity_DR7
7	Intensity_StdIntensity_Hoechst
8	Intensity_MADIntensity_Hoechst
9	Intensity_MeanIntensity_Hoechst
10	Intensity_MedianIntensity_DR7
11	AreaShape_ConvexArea
12	AreaShape_MaxFeretDiameter
13	Intensity_MedianIntensity_NeuO
14	Intensity_UpperQuartileIntensity_DR7
15	Intensity_MedianIntensity_Hoechst
16	Intensity_MADIntensity_IB4
17	Intensity_MeanIntensity_DR7
18	RadialDistribution_MeanFrac_DR7_4of4
19	Intensity_MeanIntensityEdge_IB4
20	Intensity_MeanIntensity_NeuO

Table S3. Top 20 features prioritised during Random Trees classification of cells in the screen, related to Figure 3.
DR7 = DRAQ7.

Table S5. Compounds and targets causing neurotoxicity in the absence of LPS, related to Figure 4.

Ordered by decreasing effect size. Predicted (LS) mean diff. = mean normalised neuron count after DMSO LPS- treatment minus after compound LPS- treatment. 1 represents loss of neurons equal to when treated with LPS (near 100%). #### in Name column represents (1H-Benzoimidazol-2-yl)-(3,4-dichlorobenzyl)amine. **Targets:** Gene symbols of known targets for each compound. Default on-target mechanism of action (MOA) is inhibition; suffixed * indicates agonism, # indicates binding, and ~ indicates unknown activity. Highlighted target-MOAs are those which are repeated amongst all target-MOAs of the neurotoxic compounds (e.g. SLC6A3 is inhibited by compounds 84, 73, and 125 in this list).

ID	CHEMBL ID	Predicted (LS) mean diff.	95.00% CI of diff.	Summary	Adj. p value	Name	Targets																									
219	CHEMBL429023	1.096	0.8480 to 1.343	****	<0.0001	ROTENONE	MT-ND4	MTOR	HTR6#	AR	CYBA	MT-ND1	NDUFAB1	NFE2L2																		
167	CHEMBL1870314	0.9401	0.6924 to 1.188	****	<0.0001	SID124896949	INSR#	TDP1																								
84	CHEMBL26320	0.8078	0.5601 to 1.055	****	<0.0001	Gbr-12935	SLC6A3	SIGMAR1	SLC6A2	SLC6A4	CYP2D6	CBX1	KLHL6																			
127	CHEMBL119247	0.7942	0.5465 to 1.042	****	<0.0001		NPY5R#																									
73	CHEMBL30008	0.7931	0.5454 to 1.041	****	<0.0001	Flunarizine	SIGMAR1	HRH1	ADRA2C	DRD3	SCN1A	SCN2A	SCN3A	CACNA1B	CACNA2D1	CACNB1	CYP2J2	HTR2A	DRD2	ADRA2A	SLC6A4	CHRM3	HTR2C	ADRA1A	SLC6A3	KCNH2	CACNA1G	ADRA1D	ADRA1B	HTR2B	CHRM5	CACNA1I
224	CHEMBL2093893	0.7546	0.5069 to 1.002	****	<0.0001		CACNA1C	CACNA1D	CACNA1S																							
15	CHEMBL1484	0.7311	0.4834 to 0.9788	****	<0.0001	NICARDIPINE	CACNA1C	CACNA1D	CACNA1S	CYP2C9	CYP3A4	TRPA1*																				
140	CHEMBL145	0.712	0.4643 to 0.9597	****	<0.0001	CAFFEIC ACID	MMP9	MMP2	COMT	MMP1																						
227	CHEMBL1814749	0.5252	0.2775 to 0.7729	****	<0.0001		DPP8																									
123	CHEMBL3040440	0.4979	0.2502 to 0.7456	****	<0.0001	PND-1186	PTK2	MAPK9	MAPK10																							
145	CHEMBL456444	0.4656	0.2179 to 0.7133	****	<0.0001	####	KCNN3																									
68	CHEMBL27759	0.4263	0.1786 to 0.6740	****	<0.0001	Entinostat	HDAC1	HDAC2	NCOR2	CYP3A4																						
125	CHEMBL781	0.3736	0.1259 to 0.6213	****	<0.0001	Mazindol	SLC6A2	SLC6A3	SLC6A4	SIGMAR1~	HRH2																					
126	CHEMBL2204995	0.3323	0.08461 to 0.5800	***	0.0006	GSK343	EED	EZH2	RBBP4	RBBP7	SUZ12	AEBP2	EZH1																			
143	CHEMBL101326	0.3269	0.07921 to 0.5746	***	0.0008	fipronil	GABRA1	GABRA2	GABRA3	GABRA4	GABRA5	GABRA6	GABRB1	GABRB2	GABRB3	GABRD	GABRE	GABRG1	GABRG2	GABRG3	GABRP	GABRQ										
53	CHEMBL2347651	0.2868	0.03906 to 0.5345	**	0.008	Ispinesib	KIF11																									
50	CHEMBL222102	0.2797	0.03200 to 0.5274	*	0.0115	Ku-55933	ATM	PIK3CB	PRKDC	PIK3CA																						
24	CHEMBL41	0.2752	0.02754 to 0.5229	*	0.0143	Fluoxetine	SLC6A4	ADRA2A	HTR1A*	HTR1B*	HTR1D*	HTR1F*	CYP2C19	HTR2A*	HTR2C*	SLC6A2	ACHE	NET	SIGMAR1	CYP2D6												
157	CHEMBL2424780	0.2566	0.008854 to 0.5042	*	0.0342	Vesatolimod	TLR7*																									
59	CHEMBL2396661	0.2488	0.001115 to 0.4965	*	0.0477	Alpelisib	PIK3CA	PIK3R1	PIK3CD	PIK3CG																						

Table S6. Compounds and targets affecting microglial counts in the absence of LPS, related to Figure 5.

Ordered by decreasing microglial number. Predicted (LS) mean diff. = mean normalised microglia count after DMSO LPS- treatment minus after compound LPS- treatment. -1 represents increase in microglia number equal to when treated with LPS (approximately doubling), 1 = decrease in microglia number equal to inverse of change when treated with LPS. **Targets:** Gene symbols of known targets for each compound. Default on-target mechanism of action (MOA) is inhibition; suffixed * indicates agonism and # indicates binding. Highlighted target-MOAs are those which are repeated amongst all target-MOAs of the compounds.

ID	CHEMBL ID	Predicted (LS) mean diff.	95.00% CI of diff.	Summary	Adj. p value	Name	Targets				
157	CHEMBL2424780	-2.781	-3.181 to -2.380	****	<0.0001	Vesatolimod	TLR7*				
167	CHEMBL1870314	-0.7115	-1.112 to -0.3112	****	<0.0001	SID124896949	INSR#	TDP1			
124	CHEMBL146735	-0.4537	-0.8540 to -0.05342	*	0.0109		HTR1B	HTR1D			
210	CHEMBL2103875	0.4223	0.02203 to 0.8226	*	0.0276	Trametinib	MAP2K1	MAP2K2	ABCB1		
152	CHEMBL3672369	0.4691	0.06886 to 0.8694	**	0.0066	OTS964	PBK	NEK1			
161	CHEMBL2204502	0.4866	0.08632 to 0.8869	**	0.0037	XL888	HSP90AA1	HSP90AB1			
26	CHEMBL1235110	0.496	0.09576 to 0.8963	**	0.0027		SYK	MAPK1	MAPK3	GP6	
105	CHEMBL603	0.5172	0.1170 to 0.9175	**	0.0012	Zafirlukast	CYSLTR1	CYSLTR2	MAPK14		
31	CHEMBL1451	0.5425	0.1423 to 0.9428	***	0.0005	Triamcinolone	NR3C1*	NFKB1	NFE2L2		
96	CHEMBL507361	0.5878	0.1875 to 0.9880	****	<0.0001	PD-0325901	MAP2K1	BRAF	MAP2K2	MAPK1	MAP2K5

Table S7. Compounds and targets affecting microglial counts in the presence of LPS, related to Figure 5.

Ordered by decreasing microglial number. Predicted (LS) mean diff. = mean normalised microglia count after DMSO LPS+ treatment minus after compound LPS+ treatment. -1 represents increase in microglia number twice as much as when treated with LPS (approximately doubling), 1 = decrease in microglia number equal back to LPS-untreated levels. **Targets:** Gene symbols of known targets for each compound. Default on-target mechanism of action (MOA) is inhibition; suffixed * indicates agonism and # indicates binding. Highlighted target-MOAs are those which are repeated amongst all target-MOAs of the compounds.

ID	CHEMBL ID	Predicted (LS) mean diff.	95.00% CI of diff.	Summary	Adj. p value	Name	Targets													
214	CHEMBL426184	-1.19	-1.590 to -0.7895	****	<0.0001	TAK-242/CLI-095/resatorvid	TLR4													
24	CHEMBL41	-0.9716	-1.372 to -0.5713	****	<0.0001	Fluoxetine	SLC6A4	ADRA2A	HTR1A*	HTR1B*	HTR1D*	HTR1F*	CYP2C19	HTR2A*	HTR2C*	SLC6A2	ACHE	NET	SIGMAR1	CYP2D6
111	CHEMBL707	-0.8986	-1.299 to -0.4982	****	<0.0001	Doxazosin	ADRA1B	ADRA1D	ADRA1A	SLC6A3	HTR2B	ADRA2C	HTR2C	ADRA2A	HTR4					
28	CHEMBL1358	-0.8631	-1.263 to -0.4627	****	<0.0001	Fulvestrant	PGR	ESR1	ESR2	ESRRA	EPHX2	NR1H4								
139	CHEMBL373250	-0.7039	-1.104 to -0.3035	****	<0.0001	L-838417	GABRA3*	GABRB3*	GABRG2*	GABRA2*	GABRA1*	GABRA5*								
29	CHEMBL1399	-0.6383	-1.039 to -0.2379	****	<0.0001	Anastrozole	CYP19A1													
114	CHEMBL717	-0.5941	-0.9945 to -0.1938	****	<0.0001	Medroxyprogesterone Acetate	PGR*	AR*	NR3C1*	AKR1C3	SHBG#	ESR1*	ESR2*							
128	CHEMBL1235237	-0.5466	-0.9470 to -0.1463	***	0.0004	PD004451	PDE10A													
77	CHEMBL36	-0.4958	-0.8961 to -0.09540	**	0.0027	Pyrimethamine	DHFR	SLC47A1												
5	CHEMBL139	-0.4904	-0.8908 to -0.09008	**	0.0032	Diclofenac	PTGS1	PTGS2	CXCL8	CXCR1										
79	CHEMBL385517	-0.4517	-0.8520 to -0.05132	*	0.0116	Saxagliptin	DPP4	DPP9	DPP8											
67	CHEMBL277535	-0.446	-0.8463 to -0.04560	*	0.0138	Bifonazole	CYP17A1	CYP51A1	CYP3A4											
90	CHEMBL469	-0.4419	-0.8422 to -0.04152	*	0.0157	Ketorolac	PTGS1	PTGS2	PTGDS	INHA	EPHA2									
210	CHEMBL2103875	0.4283	0.02798 to 0.8287	*	0.0233	Trametinib	MAP2K1	MAP2K2	ABCB1											
167	CHEMBL1870314	0.4376	0.03727 to 0.8380	*	0.0178	SID124896949	INSR#	TDP1												
219	CHEMBL429023	0.4682	0.06789 to 0.8686	**	0.0068	ROTENONE	MT-ND4	MTOR	HTR6#	AR	CYBA	MT-ND1	NDUFAB1	NFE2L2						
107	CHEMBL63	0.4885	0.08813 to 0.8888	**	0.0035	Rolipram	PDE4A	PDE4B	PDE4C	PDE4D										
31	CHEMBL1451	0.5402	0.1398 to 0.9405	***	0.0005	Triamcinolone	NR3C1*	NFKB1	NFE2L2											
14	CHEMBL1232461	1.118	0.6279 to 1.609	****	<0.0001		BRD4	BRD3	BRD2	BRDT										
96	CHEMBL507361	1.155	0.7547 to 1.555	****	<0.0001	PD-0325901	MAP2K1	BRAF	MAP2K2	MAPK1	MAP2K5									
161	CHEMBL2204502	1.255	0.8548 to 1.655	****	<0.0001	XL888	HSP90AA1	HSP90AB1												
152	CHEMBL3672369	1.477	1.077 to 1.877	****	<0.0001	OTS964	PBK	NEK1												
